# Gate dielectric stack design for 2D materials-based electronics

**DOI:** 10.1186/s40580-026-00546-0

**Published:** 2026-04-30

**Authors:** Minho Jin, Hojun Kim, Sejin Lee, Sangmoon Han, Ji-Yun Moon, Seungil Kim, Kyubeen Kim, Gwanwoo Kim, Gunwon Seo, Yoona Hwang, Jeongbin Lee, Sanggeun Bae, Zhihao Xu, Justin S. Kim, Soon-Gil Yoon, Jihun Mun, Jae-Hyun Lee, Min Sup Choi, Sang-Hoon Bae

**Affiliations:** 1 Department of Mechanical Engineering and Materials Science, Washington University in St. Louis, St. Louis, MO 63130 USA; 2 Research Institute for Convergence Science, Seoul National University, Seoul, 08826 Republic of Korea; 3 Department of Materials Science and Engineering, Chungnam National University, Daejeon, 34134 Republic of Korea; 4 The Institute of Materials Science and Engineering, Washington University in St. Louis, St. Louis, MO 63130 USA; 5 Division of Electronics and Electrical Engineering, Dongguk University, Seoul, 04620 Republic of Korea; 6 Advanced Instrumentation Institute, Korea Research Institute of Standards and Science, Daejeon, 34113 Republic of Korea; 7 Department of Electrical and Computer Engineering and SKKU Advanced Institute of Nanotechnology (SAINT), Sungkyunkwan University, Suwon, 16419 Republic of Korea

**Keywords:** 2D electronics, Gate dielectric, Atomic layer deposition, High-κ dielectrics, van der Waals integration

## Abstract

Two-dimensional (2D) semiconductors enable atomically thin channels and attractive electrostatics, but practical scaling increasingly hinges on gate-dielectric integration rather than channel performance. A key challenge is forming high-quality dielectrics on chemically inert, dangling-bond-free 2D surfaces while pushing equivalent oxide thickness to the sub-nanometer regime without excessive leakage, traps, or electrical breakdown. This review addresses the materials and process physics that govern dielectric formation in 2D devices, with an emphasis on atomic layer deposition nucleation, surface pretreatment and functionalization, and the use of seed and buffer layers for conformal high-κ oxides. The roles of layered insulators, such as hexagonal boron nitride, are discussed in terms of interface quality, electrostatic scaling limits, and transport limitations. The impact of dielectrics and processing on leakage mechanisms, defect generation, device-to-device variability, and reliability metrics, including time-dependent dielectric breakdown, bias-temperature instability, hysteresis, and threshold-voltage drift, is examined. Finally, we highlight van der Waals dry integration and dielectric transfer approaches that reduce process-induced damage and support wafer-scale uniformity, as well as opportunities for mixed-dimensional and 3D stacked architectures across logic, memory, and emerging functional systems.

**Graphical abstract:**

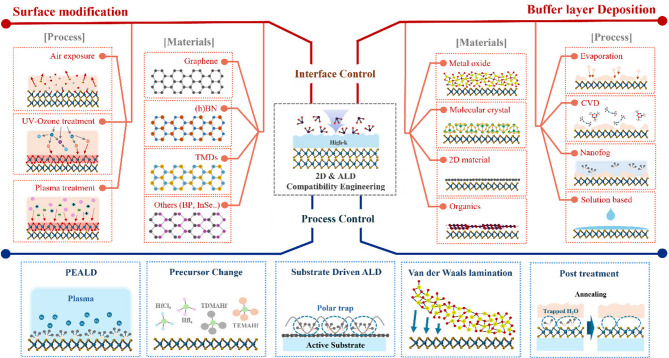

## Introduction

Two-dimensional (2D) materials enable continued transistor scaling by providing atomically thin semiconducting channels that reduce short-channel effects and enable effective electrostatic gating in nanometer- and sub-nanometer-scale devices [1, 2]. Beyond the channel itself, the defining feature of these materials is their dangling bond-free surface, which enables clean interfaces but also makes device performance particularly sensitive to extrinsic effects introduced during processing and integration [3]. This surface chemistry allows interface-engineered stacks and van der Waals (vdW) heterostructures, enabling the deterministic assembly of different crystalline materials without lattice matching, and further underscores the importance of interfacial disorder, trapped charge, and processing-induced defects for device performance and variability [4]. At the same time, translating 2D transistors to manufacturing demands more than high mobility and favorable switching characteristics, as the entire device stack must meet strict requirements for variability, yield, and reliability consistent with established silicon (Si) technologies [5]. In practice, performance in scaled, densely integrated devices is often limited by contact and interconnect parasitic, process-induced defects arising from lithography and deposition, and interfacial disorder [6]. This mismatch between materials-level potential and system-level implementation has been discussed so far, which emphasizes that 2D electronics is driven as much by integration constraints as by channel materials [7]. Accordingly, research emphasis has expanded beyond single-transistor demonstrations to heterogeneous integration strategies that incorporate 2D materials with established Si technologies and process flows [8]. Recent studies indicate that progress toward manufacturable 2D electronics requires reproducible process integration and circuit-level demonstrations with device-to-device uniformity across large device arrays [9].

A major bottleneck in the transition of 2D devices is the gate dielectric, because high-quality dielectrics must provide strong gate coupling, low leakage, low trap density, and robust breakdown margins while forming an electronically clean interface with the 2D surface [10]. Atomic layer deposition (ALD) is widely used in semiconductor technology because it provides conformal films with precise thickness control and high uniformity, making it an effective approach for integrating high-dielectric-constant (high-κ) dielectrics on atomically thin channels [11, 12]. However, ALD nucleation on 2D semiconductors is limited by a lack of surface nucleation sites, leading to discontinuous nucleation and initial growth, as well as non-ideal interfacial bonding that introduces traps and scattering [13]. While a dangling-bond-free surface can enable stable channel transport, it hinders the direct formation of conventional amorphous oxides, and these limitations become more pronounced as the equivalent oxide thickness (EOT) is scaled down. Layered-crystalline 2D insulators such as hexagonal boron nitride are often used to improve interface quality, yet quantitative studies show that their band offsets and dielectric constant (κ) constrain the achievable trade-off between leakage suppression and electrostatic scaling [14, 15]. This has led to broader exploration of dielectric materials beyond conventional oxides; yet at scaled thickness, the key constraints increasingly come from leakage physics, reliability, and variability, rather than from dielectric material itself. As physical thickness is reduced to meet EOT targets, direct tunneling and defect-assisted conduction are increasingly difficult to suppress. Under electrical stress, progressive trap generation and degradation can lead to dielectric breakdown, thereby necessitating explicit lifetime considerations and a conservative margin in device design [16]. In addition, the dielectric material affects performance beyond electrostatics. Polar dielectrics can introduce remote phonon scattering and increase self-heating, degrading mobility even with an atomically sharp interface. Stability is further limited by charge trapping in the dielectric and near-interface region, leading to hysteresis and bias-stress-induced threshold-voltage drift. The magnitude and kinetics depend on the band alignment between channel and oxide defect states, and therefore vary with device architecture and gate-stack design [17]. Importantly, at scale, these mechanisms become a source of variability because the spatial distribution of traps and breakdown precursors governs device-to-device variation, yield, and bias-induced drift in large arrays. Wafer-scale integration is also more susceptible to contamination, local nonuniformity, and defects, which can broaden these distributions and reduce reproducibility across large-area arrays [18, 19].

This review discusses how integration constraints govern dielectric-stack design in 2D electronics, focusing on ALD, nucleation on dangling bond-free surfaces, and high-κ dielectric processes on 2D materials. It also discusses vdW-based dry integration routes for dielectrics and contacts, emphasizing wafer-scale integration methods that mitigate process-induced damage and reduce variability. As continued scaling increasingly relies on vertical and heterogeneous integration, this review further discusses dielectric design in emerging 3D integration concepts and mixed-dimensional stacks enabled by vdW interfaces for vertical complementary logic. Related approaches in low-temperature monolithic stacking and printed or solution-processed 2D semiconductors highlight that dielectric deposition and treatment must remain compatible with back-end thermal budgets. Finally, dielectric integration in circuit- and system-level demonstrations is discussed, in which the gate stack must meet endurance, retention, and array-level uniformity requirements, including logic-in-memory and functional devices based on atomically thin semiconductors. Recent demonstrations of 2D in-memory devices highlight the need for dielectric stacks that sustain high current levels while maintaining low variability and stable threshold control across large arrays. These wafer-scale integration efforts for memory and neuromorphic systems further indicate that yield and device-to-device variation are strongly dominated by dielectric and interface quality.

## Basic principles of ALD and high-κ dielectrics

### Self-limiting growth mechanism and thin-film control in ALD

**Fig. 1:**
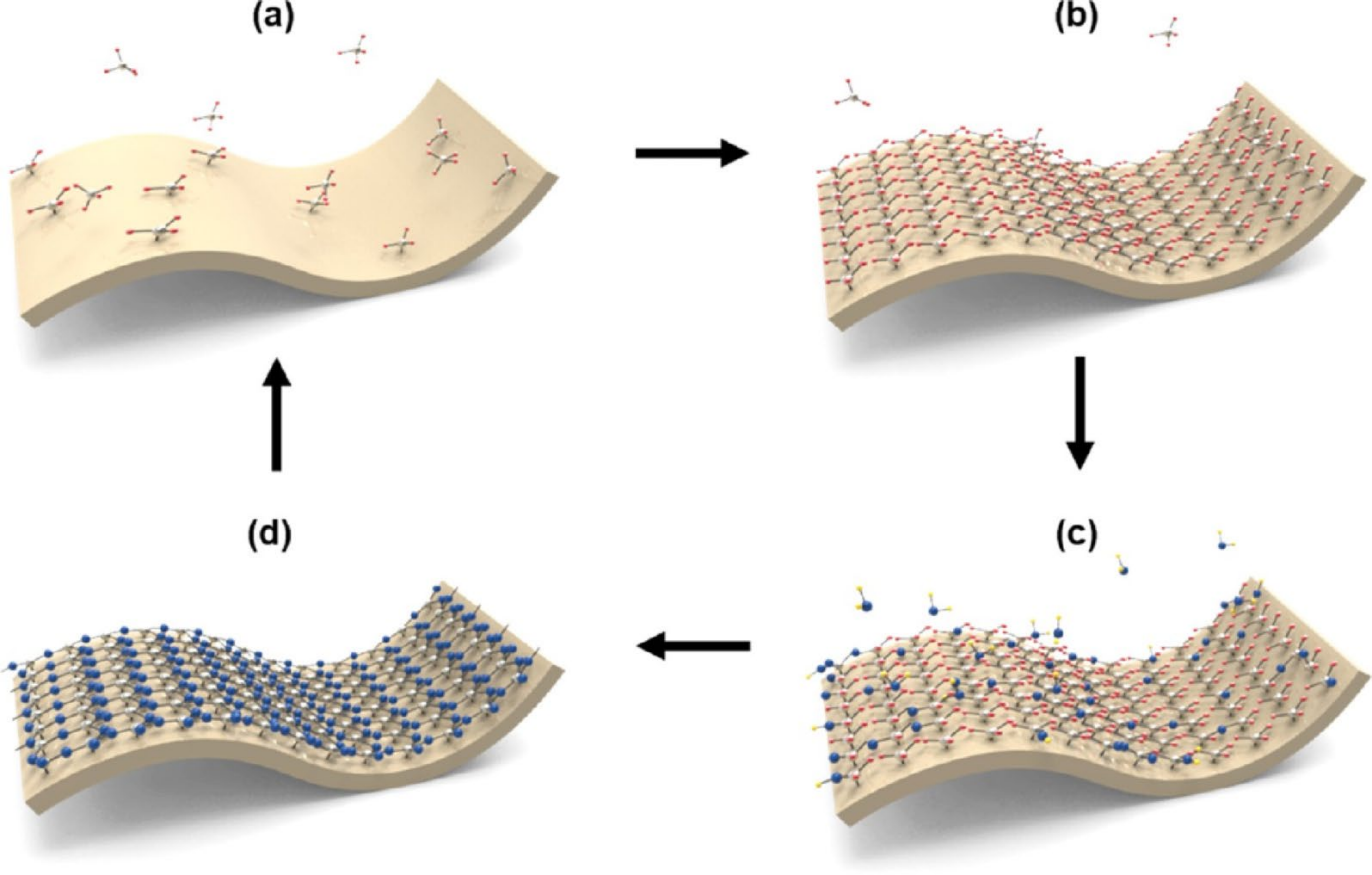
Schematic illustration of the self-limiting surface reactions during an ALD cycle. **a** Adsorption of precursor molecules on reactive surface sites. **b** Saturation of surface reactions followed by purging of excess precursor. **c** Exposure to an oxidant (or co-reactant) that reacts with the adsorbed species; and **d** removal of by-products, leading to the regeneration of reactive surface sites [20]

ALD is a widely employed deposition technique in semiconductor manufacturing, particularly for forming high-κ gate dielectrics. The process proceeds by sequentially introducing precursor and reactant gases in a cyclic manner, with surface reactions occurring exclusively at active sites and terminating spontaneously upon saturation. This self-limiting reaction mechanism enables precise control of film thickness at the atomic-layer level and allows for the formation of highly uniform thin films through repeated deposition cycles [20, 21]. A typical ALD cycle consists of four sequential steps. First, a metal precursor is introduced and chemisorbs onto the substrate surface. Once the surface reaches saturation, the adsorption reaction naturally terminates. This is followed by a purge step to remove excess precursor molecules and reaction byproducts from the chamber. Subsequently, a reactant gas, such as an oxidant or nitridant, is introduced to react with the adsorbed precursor species. Finally, a second purge step removes the remaining reaction byproducts, completing one ALD cycle [13, 22, 23]. By repeating this cycle tens to hundreds of times, dielectric films with the desired thickness can be precisely formed [20] (Fig. 1).

Compared to other deposition techniques, ALD offers several key advantages. Most notably, film thickness can be controlled with sub-Å precision, enabling excellent thickness uniformity and reproducibility [20, 24–27]. In addition, ALD typically operates at relatively low process temperatures (generally 200–300 °C), making it highly compatible with thermally sensitive substrates and materials. Moreover, ALD ensures outstanding conformality even on high-aspect-ratio structures, which is critical for advanced transistor architectures such as Fin field-effect transistors (FinFETs) and gate-all-around (GAA) devices [24, 27–29]. As a result, ALD has emerged as an effective alternative to conventional chemical vapor deposition (CVD) and physical vapor deposition (PVD) techniques, which often suffer from film nonuniformity, limited thickness control, and increased interfacial defect formation.

Beyond these structural advantages, ALD also demonstrates superior performance in reducing interfacial trap density (*D*_it_), improving thickness reproducibility, and enhancing electrical reliability compared to CVD and PVD processes [20, 25, 28–32]. These characteristics are particularly critical for 2D semiconductor devices, where interfacial quality directly governs device performance. Because 2D materials are chemically inert and exhibit low intrinsic surface reactivity, precursor adsorption and nucleation during ALD are highly sensitive to surface charge states, Fermi level position, and interfacial defect density [33, 34]. Furthermore, the physical origin of ALD nucleation on dangling-bond-free 2D surfaces must also be considered. Because the basal plane provides few reactive adsorption sites, nucleation typically initiates at defect-rich regions, such as edges, grain boundaries, wrinkles, cracks, and vacancies [35, 36]. These sites exhibit enhanced reactivity due to locally perturbed bonding configurations and electronic structures, thereby lowering adsorption barriers and facilitating precursor binding [37, 38]. Accordingly, surface activation, functionalization, and seed-layer engineering serve to introduce or extend reactive sites beyond these intrinsic defects, enabling more uniform dielectric growth across the 2D surface. Consequently, careful selection of precursor chemistry and process conditions tailored to the substrate doping level and oxidation state is essential. Such control over surface–chemical interactions forms the foundation for surface modification and seed-layer engineering strategies, which are discussed in the following sections [20, 27].

### Limitations of hBN for 2D FETs

In devices based on 2D semiconducting transition metal dichalcogenides (TMDs), the gate dielectric is not merely an insulating layer but a key determinant of both electrical performance and long-term reliability [10, 24, 28, 39]. An ideal dielectric should therefore possess a high-κ to enable scaling of EOT below 1 nm while simultaneously suppressing leakage current, and it must provide sufficiently large band offsets with respect to the channel [13, 24, 40, 41]. In general, a stable gate insulator requires an energy difference in conduction- and valence-band offsets exceeding ~ 1.5 eV. In addition, chemical stability is essential to prevent interfacial reactions or oxidation during processing, and *D*_it_ must be minimized to avoid charge trapping, hysteresis, and threshold-voltage instability [10, 17, 28, 40–42].

**Fig. 2:**
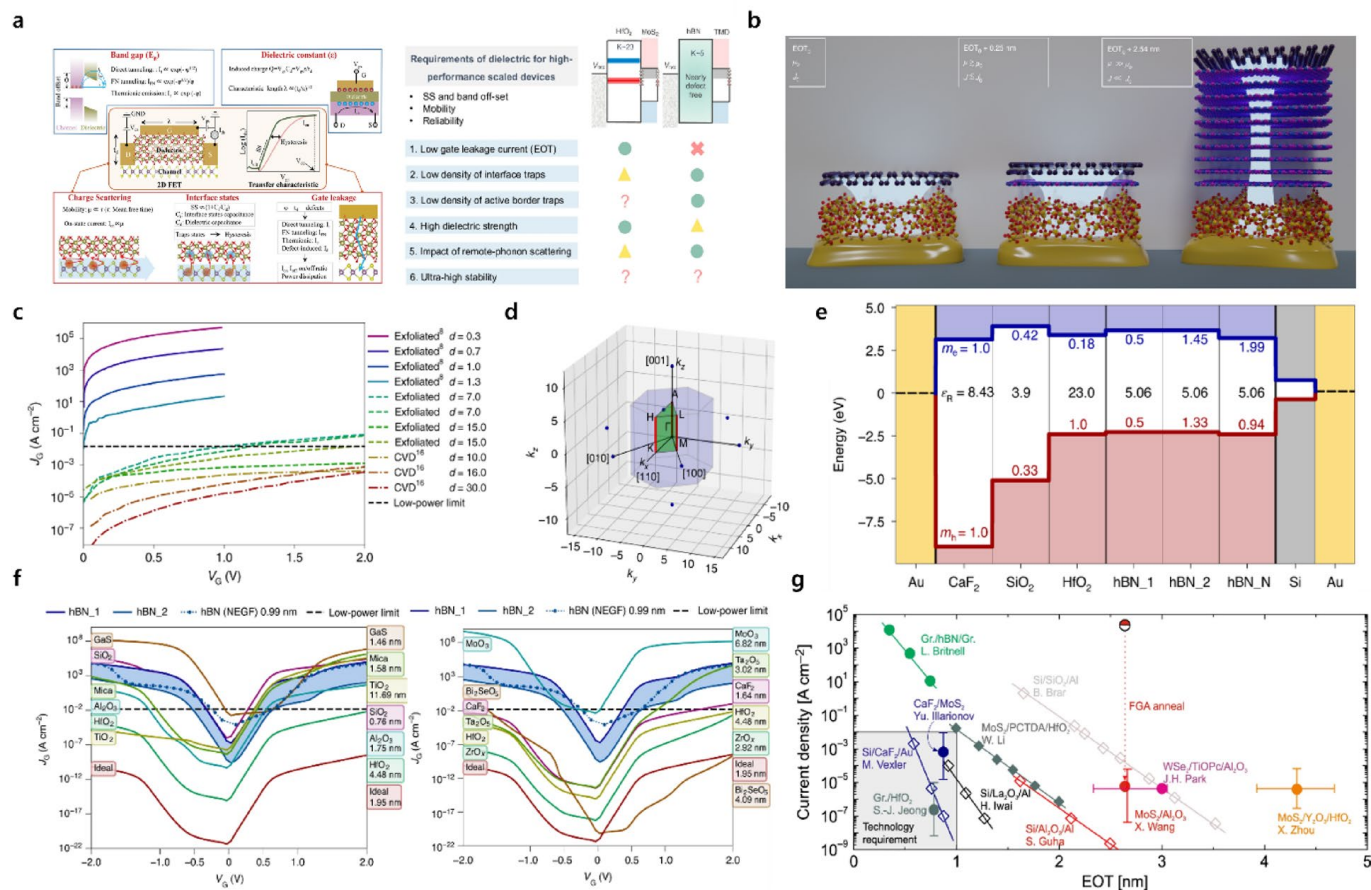
**a** (Left) Device performance of a 2D FET strongly depends on the gate dielectric layer. (Right) Comparison between high-κ HfO_2_ and crystalline hBN dielectric for scaled FETs [28, 40]. **b** Impact of hBN interlayers on current density through the insulating stack [14]. **c** Preparation methods of hBN and thickness-dependent leakage current, where ${\boldsymbol{d}}\boldsymbol{ }$denotes the hBN thickness in nanometers [14]. **d** Brillouin zone and **e** band structure of various dielectrics, including hBN [14]. **f** Comparison of calculated gate leakage currents for hBN and other dielectrics under identical EOT conditions. (left: 3D amorphous and 2D layered dielectrics; right: native oxides and ionic insulators) [14]. **g** Relationship between gate leakage current density and EOT extracted from various 2D FET technologies [10]

Figure 2a schematically summarizes these key requirements for gate dielectrics in highly scaled 2D FETs, highlighting the need for both superior interfacial quality and robust electrostatic control [28, 40]. More recently, the ability of a dielectric to stabilize the Fermi level of the channel has emerged as an important metric. In particular, for n-type TMD channels, unintentional p-type doping can be induced depending on the oxygen affinity or residual charge states at the dielectric surface [6, 17, 28, 40, 42]. Such effects directly influence band alignment and field-effect control, and thus must be carefully considered in dielectric selection. Representative high-κ dielectrics that satisfy many of these requirements include HfO_2_, ZrO_2_, and Al_2_O_3_. These materials offer high-κ values and wide band gaps (> 5 eV), are compatible with ALD processing, and can form relatively stable interfaces with 2D channels [6, 10, 40, 42, 43]. While these high-κ oxides provide excellent electrostatic control and scalability, their interfaces with 2D channels are often characterized by significant interfacial disorder and a substantial population of dangling bond-derived trap states [44].

From the perspective of forming an ideal interface with 2D semiconductors, hexagonal boron nitride (hBN) has attracted significant attention. Studies employing hBN as a gate dielectric or interfacial passivation layer have demonstrated improved threshold-voltage stability, enhanced long-term reliability, and better device-to-device uniformity [6, 10, 28, 40, 42]. Nevertheless, the intrinsically low dielectric constant of hBN imposes severe limitations on EOT scaling. As illustrated in Fig. 2b, increasing the thickness of the hBN interlayer leads to a larger EOT, thereby fundamentally altering gate electrostatics [14]. While thicker hBN can suppress Coulomb and remote-phonon scattering, thereby improving channel mobility, the concomitant increase in EOT reduces gate capacitance and weakens electric-field coupling. This trade-off can be understood more quantitatively. For instance, WSe_2_ devices fabricated on 2D hBN substrates exhibit significantly higher carrier mobilities (56–121 cm^2^ V^−1^ s^−1^) compared to those on conventional SiO_2_/Si substrates (2–21 cm^2^ V^−1^ s^−1^), underscoring the transport advantage provided by the atomically clean hBN interface [45]. In contrast, the out-of-plane dielectric constant of hBN remains relatively low, in the range of ~ 3.29–3.76 depending on thickness, which fundamentally limits gate capacitance and EOT scaling [46]. Furthermore, prior studies suggest that an hBN interlayer thickness exceeding ~ 3.3 nm is required to effectively suppress carrier scattering, corresponding to EOT values greater than ~ 2.5 nm [14]. Therefore, although increasing the hBN thickness enhances carrier transport, it simultaneously incurs a substantial electrostatic penalty, limiting EOT values. Accordingly, hBN should be regarded primarily as a mobility-preserving interfacial layer rather than a standalone gate dielectric for aggressively scaled logic devices. Moreover, as shown in Fig. 2c, the magnitude of the leakage current exhibits a strong dependence on both the thickness of the hBN layer and the specific growth conditions employed for hBN synthesis. Under aggressively scaled complementary metal–oxide–semiconductor (CMOS) operating conditions, this loss of electrostatic control hampers effective suppression of gate leakage and degrades switching behavior. These limitations originate from the electronic structure and band alignment of hBN. Figure 2d presents calculated band structures of hBN, showing that despite its large band gap, multiple k-space pathways contribute to conduction, and both electrons and holes exhibit relatively small effective masses, which is unfavorable for tunneling suppression. This behavior is further corroborated by the band-alignment comparison in Fig. 2e, owing to its low dielectric constant. hBN is constrained to thin physical thicknesses at a given EOT, and the combination of a relatively high valence-band position and a small effective mass makes it particularly difficult to achieve a sufficient tunneling barrier under pMOS operating conditions.

These intrinsic material limitations are difficult to overcome even in the absence of defects. Indeed, Fig. 2f, based on calculations assuming defect-free hBN, shows that in the ultrathin EOT regime, gate leakage through hBN is substantially higher than that through high-κ oxides or ionic insulators. Furthermore, Fig. 2g compiles experimental data from various 2D FET technologies, demonstrating that, despite excellent interfacial quality, hBN-based structures struggle to meet the stringent low-leakage requirements in the deeply scaled EOT regime [10, 28]. To place these competing effects on a quantitative basis, consider the series-capacitance model for a composite hBN/high-κ gate stack. The total EOT is the sum of contributions from each layer, EOT = EOT_hBN_ + EOT_high-κ_. Because κ_hBN_ ≈ 3–4, even a single monolayer of hBN (≈0.33 nm) adds ≈0.32–0.43 nm to the total EOT, and a bilayer (≈0.66 nm) contributes ≈0.64–0.86 nm. For comparison, the same physical thickness of HfO_2_ (κ ≈ 20–25) would contribute only ≈0.05–0.06 nm to EOT. In devices targeting sub-1 nm EOT, an hBN interlayer therefore consumes a substantial fraction of the available EOT budget before the high-κ layer is even considered. On the mobility side, however, the benefits of hBN are substantial. hBN-encapsulated monolayer MoS_2_ devices have demonstrated Hall mobilities approaching 150–200 cm^2^·V^−1^ s^−1^ at room temperature, nearly an order of magnitude higher than the 20–40 cm^2^·V^−1^·s^−1^ values typically observed on SiO_2_ [47]. This mobility improvement originates from the suppression of Coulomb scattering by charged impurities and the elimination of remote surface optical phonon modes that are prominent in amorphous SiO_2_ and high-κ oxides [48]. Yet the quantitative analysis is clear that at sub-1-nm EOT targets, even monolayer hBN already contributes ≥ 30% of the total EOT budget, severely limiting the thickness available for the high-κ component and thereby constraining achievable gate capacitance and on-current density. The leakage is equally significant, as theoretical calculations assuming defect-free hBN predict that, at an EOT of 1 nm, the gate leakage current density through hBN exceeds 10^2^ A·cm^–2^, several orders of magnitude above the low-power CMOS limit of ∼10^−2^ A cm^−2^, owing to the relatively small carrier effective masses and modest band offsets of hBN. In contrast, HfO_2_ at the same EOT exhibits leakage below 10^−2^ A·cm^−2^ due to its much larger thickness and heavier tunneling effective masses [14]. This quantitative comparison reveals an inherent trade-off, in that hBN is essential for achieving high channel mobility and low interface trap density. Still, its inclusion inevitably compromises electrostatic scaling and leakage performance. Optimal gate-stack design must therefore explicitly balance mobility gain against EOT disadvantages on a per-application basis. For these reasons, hBN is more effective as an auxiliary interfacial modifier rather than as a standalone gate dielectric. A representative strategy is a composite gate stack in which a thin hBN layer is inserted on the channel, followed by ALD deposition of a high-κ dielectric. Such architectures leverage the intrinsically low *D*_it_ of hBN, together with the high capacitance of high-κ oxides, enabling simultaneous interfacial stability, Fermi-level preservation, and leakage-current suppression [10, 13, 28, 42]. Ultimately, in 2D semiconductor devices, the ideal dielectric is unlikely to be realized as a single material; instead, a composite approach that simultaneously ensures chemical interfacial stability and strong electrostatic control is required. The following sections, therefore, discuss strategies for implementing composite structures, including surface modification, seed-layer insertion, and ALD process optimization.

## Strategies for enhancing dielectric deposition on 2D TMDs

### Early research and nucleation issues on 2D surfaces

2D materials possess atomically thin layered structures held together by vdW interactions and inherently lack dangling bonds on their surfaces [49, 50]. This chemically inert surface characteristic leads to insufficient reactions with ALD precursors during deposition [51], resulting in delayed oxide nucleation and difficulty achieving uniform, continuous thin films. As a consequence, oxide layers tend to grow in an island-like morphology or form discontinuous films during the initial growth stage, rather than uniform continuous layers [23, 52]. When such nonuniform oxides form on 2D materials, nanoscale pinholes inevitably appear within the dielectric, leading to increased leakage currents that degrade device performance and compromise reliability. Therefore, achieving uniform, pinhole-free oxide thin films on 2D materials is a critical challenge in the fabrication of 2D electronic devices.

**Fig. 3:**
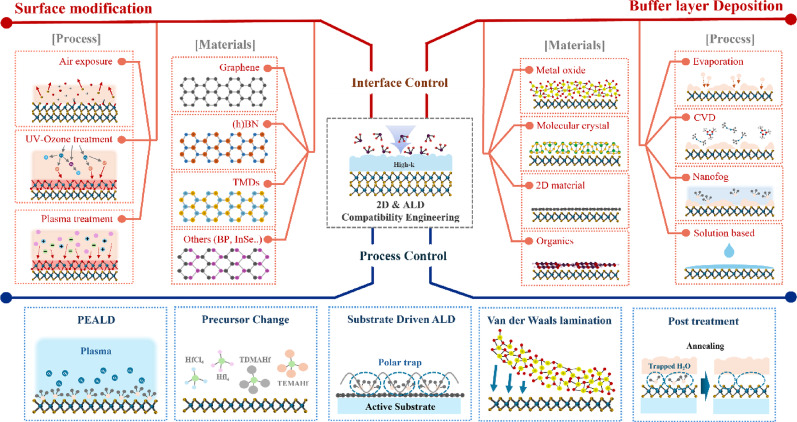
A schematic illustration of ALD-based high-κ dielectric deposition on 2D materials

To address these issues, extensive efforts have been devoted to introducing pretreatment processes before ALD to directly modify the surfaces of 2D materials and create reactive sites that interact with ALD precursors. These approaches can be broadly classified into two categories, as illustrated in Fig. 3 One involves surface modification techniques that enhance surface reactivity by introducing defects or forming additional oxide species through plasma or ozone treatments. The other employs buffer layers, in which polymers or metal oxides are deposited on the 2D material surface to promote nucleation. Beyond these approaches, process control strategies are also introduced, including modifications to ALD parameters (e.g., precursors and reactants) and indirect deposition schemes in which dielectrics are first grown on sacrificial substrates and subsequently transferred onto 2D materials, thereby avoiding direct damage to the channel. Collectively, these studies have significantly advanced the understanding of ALD nucleation on 2D materials, highlighting that deliberate interfacial engineering is indispensable for achieving uniform, high-quality oxide layers.

### Surface modification

#### NO_2_ +TMA

To achieve direct surface modification of 2D materials, a chemically induced functionalization approach using NO_2_ and trimethylaluminum (TMA) has been proposed. Farmer et al. attempted to create nucleation sites on graphene before Al_2_O_3_ deposition by using TMA and NO_2_ as reactants [53]. However, when NO_2_ is used alone, materials with high curvature or low surface energy—such as carbon nanotubes—tend to suffer from facile NO_2_ desorption during the purge step, leading to nonuniform initial nucleation. To overcome this limitation, Wang et al. introduced a NO_2_/TMA pretreatment-based chemical functionalization strategy [54]. In this approach, NO_2_ acts as an electron acceptor and adsorbs onto the sp^2^ carbon surface, after which TMA reacts with the adsorbed NO_2_ to induce stable surface modification. This synergistic interaction enables uniform thin-film deposition by providing chemically stable reactive sites. Subsequently, Young et al. applied five cycles of NO_2_/TMA pretreatment to highly ordered pyrolytic graphite (HOPG), forming reactive sites with an effective thickness of approximately 5.7 Å [55]. Following this pretreatment, high-quality Al_2_O_3_ films with high charge-transfer resistance were achieved using only 20 ALD cycles of the TMA/H_2_O process. In contrast, approximately 100 ALD cycles without pretreatment were required to obtain comparable film quality. Notably, this approach enables uniform deposition at a relatively low temperature of 150 °C with a reduced process time, while exerting minimal influence on the electrical properties of the underlying 2D material. As such, the NO_2_/TMA pretreatment strategy represents an efficient and effective method for surface modification of 2D materials.

#### Plasma treatments

Plasma treatment is one of the long-established and widely used methods for surface modification of 2D materials, as energetic species such as ions and electrons interact with the substrate surface to form new functional groups [56, 57]. In particular, oxygen plasma partially oxidizes 2D materials, forming an ultrathin oxide layer on their surfaces, thereby activating otherwise chemically inert surfaces and providing reactive nucleation sites that enable uniform dielectric growth during ALD. Oxygen-plasma-based pretreatments have therefore been widely employed to achieve uniform deposition of high-κ dielectrics, such as HfO_2_, on various TMDs with MX_2_ structures, including MoS_2_, WSe_2_, and MoTe_2_. During this process, oxygen radicals and ions generated in the plasma break surface bonds of the 2D material and substitute chalcogen atoms, forming surface oxides (MO_x_), which promote more effective reactions between ALD precursors and the TMD surface [58, 59].

ALD growth behavior of Al_2_O_3_ and HfO_2_ on multilayer MoS_2_ surfaces with oxygen plasma treatment has been systematically investigated [60]. In this study, an inductively coupled plasma source was employed to spatially separate the plasma generation region from the treatment region, thereby reducing direct plasma-induced damage. Multilayer MoS_2_ flakes prepared by mechanical exfoliation were used, and thermal ALD of Al_2_O_3_ and HfO_2_ was carried out at 250 °C. In the absence of plasma pretreatment, island formation and pinholes were observed during the initial ALD growth stage, and the surface coverage was limited to only ~ 23%. As the film thickness increased to 10 nm and 30 nm, the coverage improved to 77% and 97%, respectively; however, island-like nonuniform growth persisted. In contrast, after a 10 s oxygen plasma pretreatment, the film coverage increased from 77 to 93%, and extending the treatment time to 30 s almost eliminated island formation. These results indicate that even a short plasma exposure (~ 10 s) is sufficient to activate the MoS_2_ surface and generate adequate adsorption sites required for precursor reactions. Atomic force microscopy (AFM) measurements further revealed extremely smooth surfaces, with RMS roughness values of 0.07 nm for Al_2_O_3_ and 0.18 nm for HfO_2_ films. Despite these advantages, oxygen plasma treatment has intrinsic limitations, as high-energy ions and reactive oxygen species can induce surface oxidation and structural damage in 2D channel materials, thereby degrading device performance [61]. This issue becomes particularly severe for atomically thin TMDs. To mitigate plasma-induced damage, alternative approaches, such as remote plasma configurations or low-damage pretreatments with H_2_O plasma rather than oxygen plasma, have been proposed. Similarly, for MoTe_2_ channels subjected to O_2_ plasma pretreatment, increasing the pretreatment duration from 10 to 30 s led to a pronounced reduction in the surface roughness of the ALD-grown HfO_2_ film, from 0.595 nm to 0.352 nm. Notably, this roughness is notably lower than that of HfO_2_ deposited by plasma-enhanced ALD (PEALD), which exhibits an RMS value of 0.781 nm [62].

**Fig. 4:**
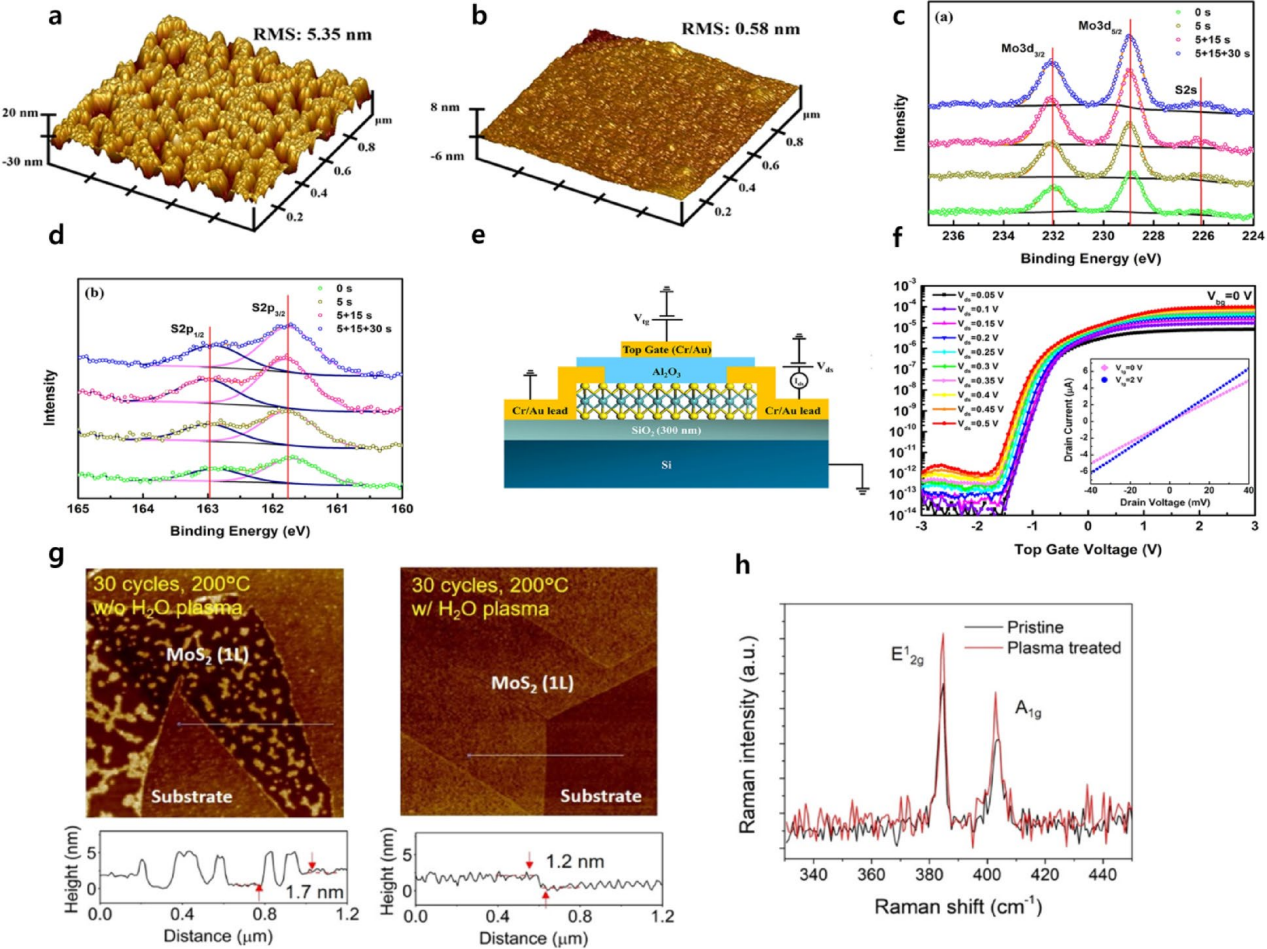
**a** AFM image of Al_2_O_3_ directly deposited on a MoS_2_ without surface pretreatment, showing non-uniform nucleation and island-like growth. **b** AFM image of Al_2_O_3_ grown on a 2D material after O_2_ plasma pretreatment, demonstrating increased nucleation density and the formation of a more continuous and uniform dielectric film. XPS spectra of MoS_2_ as a function of O_2_ plasma treatment time for **c** Mo 3d and S 2 s core levels and **d** S 2p core levels [52]. **e** Cross-sectional schematic of the top-gate few-layer MoS_2_ transistor. **f** Transfer characteristics of the top-gate few-layer MoS_2_ transistor, with the inset showing output curves at top-gate voltages of 0 and 2 V. AFM images after 30 cycles of TMA/H_2_O ALD at 200 °C on **g** (left) pristine MoS_2_ and (right) MoS_2_ treated with H_2_O plasma for 10 min. **h** Raman spectra of pristine and H_2_O plasma-treated MoS_2_ [63]

Yang et al. performed oxygen plasma pretreatment before Al_2_O_3_ deposition to form uniform dielectric films on MoS_2_ and subsequently fabricated top-gated FETs [52]. As shown in Fig. 4a, b, Al_2_O_3_ grown without plasma pretreatment exhibited island-like morphology with pinholes, whereas uniform and continuous films were obtained on MoS_2_ after remote plasma pretreatment. X-ray photoelectron spectroscopy (XPS) and Raman spectroscopy showed no detectable peak shifts after plasma treatment (Fig. 4c, d), confirming that MoS_2_ was not oxidized and that oxygen species were merely adsorbed on the surface, serving as reaction sites for ALD precursors. Electrical characterization of the top-gated FETs demonstrated excellent device performance despite the ultrathin Al_2_O_3_ thickness (~ 6.6 nm), including a low leakage current density of ~ 0.1 pA µm^−2^ at an electric field of 4.5 MV cm^−1^, an on/off current ratio exceeding 10^8^, a carrier mobility of 28 cm^2^ V^−1^ s^−1^, and a subthreshold swing (SS) of 101 mV dec^−1^ (Fig. 4e, f).

To further address channel surface oxidation associated with oxygen plasma or Ultraviolet ozone (UV–O_3_) treatments, Huang et al. proposed an H_2_O plasma pretreatment performed for 30 s before ALD to enable uniform Al_2_O_3_ deposition. H_2_O plasma introduces surface –OH groups, which suppress oxidation of MoS_2_ while simultaneously serving as nucleation sites that promote chemical adsorption of ALD precursors. As a result, highly uniform Al_2_O_3_ films with an RMS roughness of 0.16 nm were achieved (Fig. 4g) [63]. Raman spectra acquired before and after H_2_O plasma treatment showed no peak shifts (Fig. 4h), confirming the absence of oxidation and demonstrating that continuous dielectric films can be obtained even on monolayer MoS_2_.

#### UV–O_3_ treatment

**Fig. 5:**
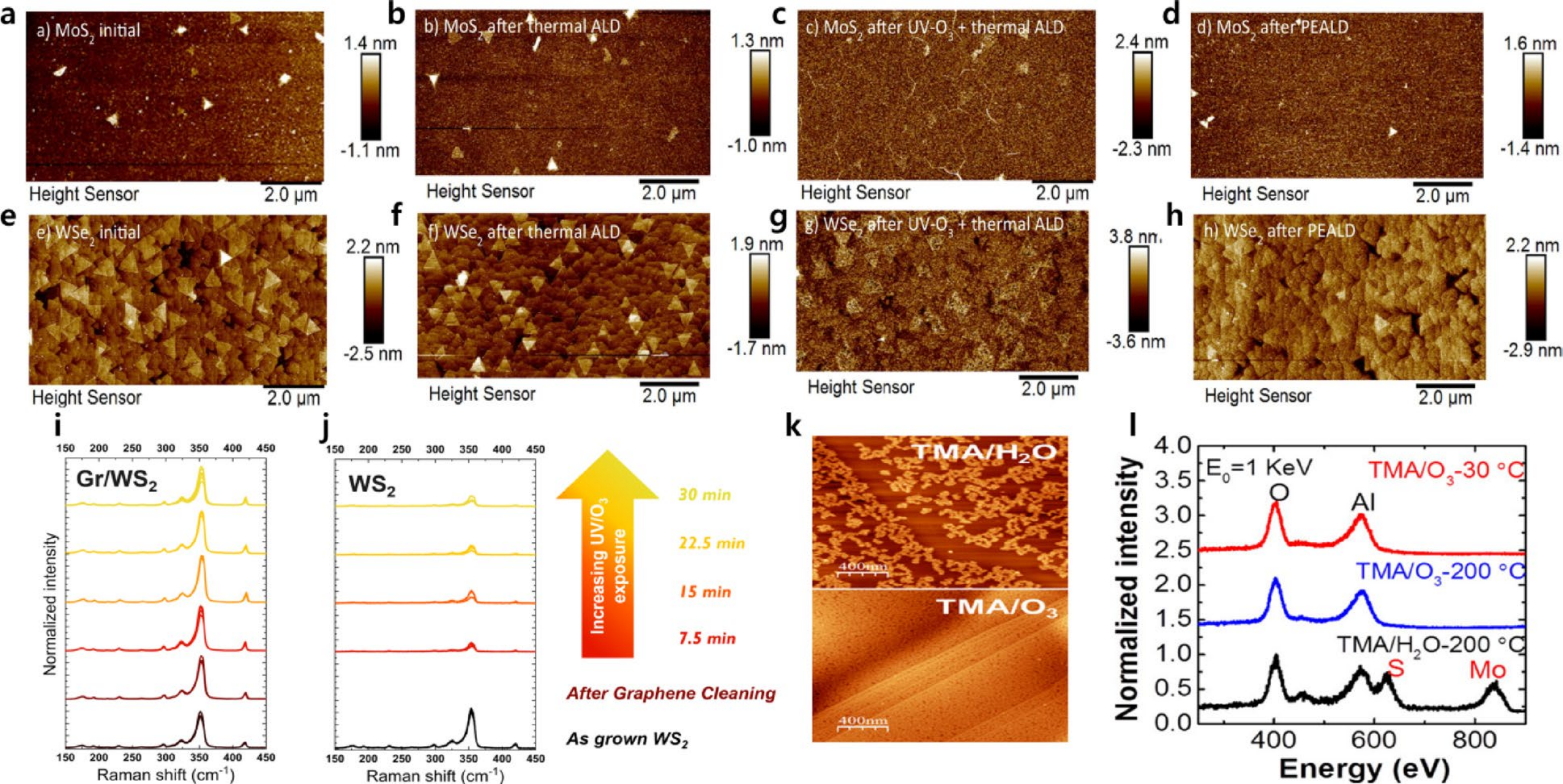
AFM images of MoS_2_ samples: **a** before ALD, **b** after thermal ALD, **c** after UV–O_3_ + thermal ALD, and **d** after PEALD. AFM images of WSe_2_ samples: **e** before, **f** after thermal ALD, **g** after UV–O_3_ + thermal ALD, and **h** after PEALD [65]. **i** Raman spectra of UV–O_3_ exposed WS_2_ with graphene capping, and **j** uncapped WS_2_ [66]. **k** AFM images of the ALD-grown Al_2_O_3_ films deposited using TMA/H_2_O (30 cycles) and TMA/O_3_ (30 cycles) at 200 °C [64]. **l** LEIS spectra of the topmost layers of ALD Al_2_O_3_ films deposited using TMA/O_3_ (30 cycles) at 30 and 200 °C, as well as TMA/H_2_O (50 cycles) at 200 °C [64]

UV–O_3_ treatment has been employed as an effective process for activating and modifying the surfaces of 2D materials, exhibiting functional similarities to oxygen plasma treatments. Upon UV irradiation, molecular oxygen dissociates into highly reactive atomic oxygen and ozone species, which subsequently interact with the surface to generate various reactive sites [64]. Walter et al. deposited ZnO thin films on MoS_2_ and WSe_2_ surfaces using ALD following UV–O_3_ pretreatment, as shown in Fig. 5a–h [65]. When thermal ALD was performed without pretreatment, negligible ZnO growth was observed on TMD surfaces even after 500 ALD cycles (Fig. 5b, f)[65]. In contrast, after UV–O_3_ pretreatment, uniform ZnO growth was achieved on MoS_2_ after approximately 60 ALD cycles (Fig. 5c). For WSe_2_, however, ZnO growth was limited to localized island formation near edges and defect sites (Fig. 5g). This material-dependent behavior was attributed to differences in the energetics of oxygen adsorption and the desorption activation barriers. Specifically, MoS_2_ exhibits a greater energy reduction upon oxygen adsorption and a higher desorption barrier than WSe_2_, resulting in more effective surface modification, whereas such reactions are less energetically favorable on WSe_2_ surfaces.

Wyndaele et al. demonstrated a graphene-mediated UV–O_3_ strategy for depositing ALD HfO_2_ on monolayer WS_2_ [66]. In this approach, graphene was first transferred onto a monolayer of WS_2_, followed by UV–O_3_ treatment to selectively oxidize the top graphene layer. Here, graphene served a dual role as a passivation layer protecting the underlying monolayer WS_2_ and as an intermediary layer facilitating ALD nucleation. After UV–O_3_ exposure, the top graphene layer was converted into a thin graphene oxide (GrO) layer, which effectively promoted uniform nucleation of HfO_2_. Notably, Raman spectroscopy revealed that graphene-capped monolayer WS_2_ exhibited negligible peak changes even after 30 min of UV–O_3_ treatment, indicating strong suppression of oxidation (Fig. 5i, j) [66]. In contrast, uncapped WS_2_ underwent rapid oxidation within 7.5 min, with WS_2_ Raman peaks almost completely disappearing. These results demonstrate that transferring graphene onto monolayer TMDs before UV–O_3_ treatment enables uniform HfO_2_ nucleation without inducing surface damage. For Al_2_O_3_, Cheng et al. have performed ozone-based ALD to compare TMA + O_3_ process with TMA + H_2_O process, as shown in Fig. 5k [64]. More uniform Al_2_O_3_ films with a thickness of 3.1 nm were obtained using an ozone-based process. Low-energy ion scattering (LEIS) measurements also revealed a higher surface coverage for the TMA + O_3_ process than for the TMA + H_2_O process, consistent with AFM observations (Fig. 5l).

### Buffer layer

In addition to approaches that directly modify the surface of 2D materials, an alternative strategy is to introduce an external layer to promote the adsorption of ALD precursors and improve the quality of dielectric thin films. The thin initial film introduced in this manner is commonly referred to as a buffer layer, which tunes the surface condition to enable uniform nucleation. Buffer-layer materials can be broadly categorized into metal-based oxides, non-metal inorganic oxides, and organic layers. These buffer layers should be rich in functional groups that can react with the precursor species, and they are typically required to be ultrathin to minimize the reduction in the effective dielectric constant due to the series-capacitor effect.

#### Metal-oxide-based buffer layers

**Fig. 6:**
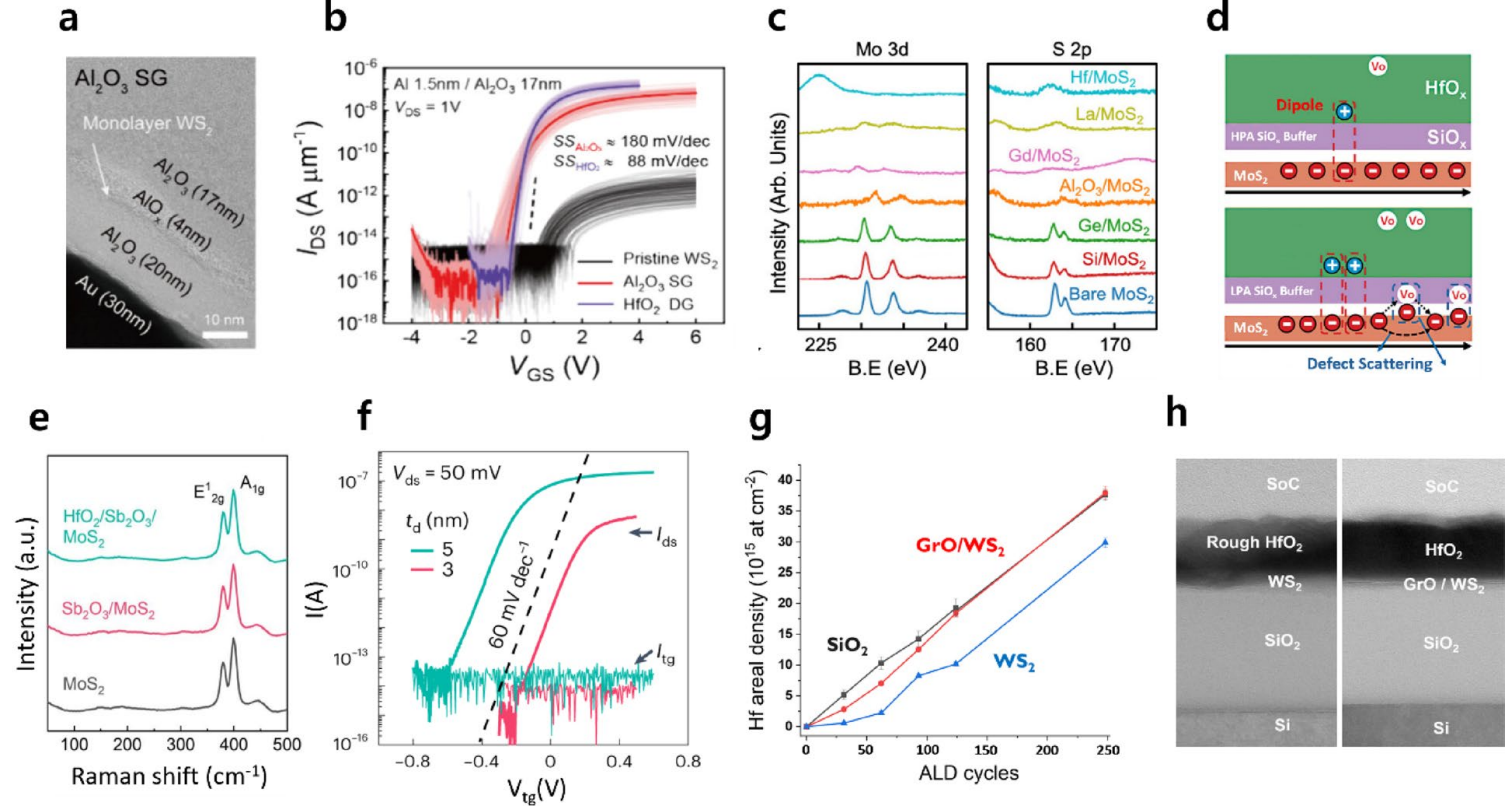
Buffer layer assisted ALD. a. Cross-sectional TEM images of single-gate (SG) WS_2_ FETs incorporating **a** AlO_x_ buffer layer [77]. **b** Transfer characteristics of HfO_2_ dual-gate (DG) devices (purple), Al_2_O_3_ SG devices with optimal doping (red), and Al_2_O_3_ SG devices without doping (black) [77]. **c** XPS spectra for monolayer MoS_2_ upon deposition of various buffer layers with an identical thickness of 2 nm [98]. **d** Schematic illustrations of carrier transport mechanisms in devices, employing SiO_x_ buffer layers formed by high-pressure annealing (HPA, top) and low-pressure annealing (LPA, bottom) [99]. **e** Raman spectra of exfoliated MoS_2_ before and after the deposition of Sb_2_O_3_ and HfO_2_ [101]. **f** Transfer characteristics of monolayer MoS_2_ FETs with dielectric thicknesses (*t*_d_) of 3 nm (red, 1 nm Sb_2_O_3_ + 2 nm HfO_2_) and 5 nm (cyan, 1 nm Sb_2_O_3_ + 4 nm HfO_2_). **g** Rutherford backscattering spectroscopy analysis, showing the change in Hf areal density on WS_2_ with and without the GrO buffer layer [66]. **h** STEM images of HfO_2_ films deposited (left) on pristine WS_2_ and (right) on GrO/WS_2_ heterostructures [66]

Among metal-oxide-based buffer layers, aluminum oxide (AlO_x_) seeding layers are the most widely adopted. In particular, the natural oxidation of ultrathin Al layers deposited via thermal or electron-beam evaporation has been extensively employed by many research groups, and this strategy has been applied not only to conventional FETs but also to a wide range of functional devices, including photonic devices, floating-gate memories, and resistive random-access memory (RRAM) [67–76]. In a representative study by Yang et al., naturally oxidized AlO_x_ was employed as a top-gate buffer layer on WS_2_ [77]. Transmission electron microscopy (TEM) images revealed the formation of an approximately 4 nm-thick AlO_x_ buffer layer on monolayer WS_2_, on top of which a uniform Al_2_O_3_ dielectric film was grown, resulting in excellent electrical characteristics (Fig. 6a, b).

Beyond natural oxidation, AlO_x_ buffer layers have also been formed directly through electron-beam evaporation (EBE) or ALD processes, and low-temperature ALD-AlO_x_ has been utilized to induce HfO_2_ growth on inert surfaces such as HOPG [78–82]. Notably, Zhang et al. demonstrated that 10 ALD cycles of Al_2_O_3_ could uniformly cover HOPG without any surface pretreatment, enabling its use as an effective buffer layer for subsequent HfO_2_ deposition [82]. The insertion of ALD-grown AlO_x_ buffer layers has also been shown to provide stability benefits by suppressing TMD oxidation. Specifically, when a 5 nm-thick ALD AlO_x_ buffer layer was inserted before the deposition of a 2.5 nm HfO_2_ layer by PEALD, the Mo^6+^/Mo^4+^ intensity ratio decreased to 0.22, compared to 0.75 for devices with direct HfO_2_ deposition, thereby demonstrating effective suppression of TMD oxidation [83].

Nevertheless, due to material-dependent differences in surface reactivity among 2D materials, additional pretreatment steps, such as UV–O_3_ exposure, may be required to ensure uniform buffer-layer deposition [84, 85]. As an alternative approach, low-temperature hybrid buffer formation based on the nanofog method, where CVD-like components are intentionally induced by controlling purge times during ALD, has also been proposed [84, 85]. Ko et al. employed the nanofog approach to form an AlO_x_ buffer layer at 50 °C before HfO_2_ deposition on MoS_2_ [86]. While devices incorporating evaporated Al buffers exhibited reduced Raman peak intensities and pronounced n-type doping, the nanofog-derived buffers showed minimal changes in the E_2g_ and A_1g_ Raman modes and nearly hysteresis-free transfer characteristics. Using a ~ 2 nm-thick AlO_x_ buffer layer, a low EOT of ~ 1.3 nm and a leakage current density below 5 × 10^–8^ A·cm^−2^ were achieved, highlighting the effectiveness of the nanofog-based buffer formation strategy.

In addition to AlO_x_, a variety of metal and metal-oxide buffer layers, including TaO_x_ and Y_2_O_3_, have been reported [87–97]. Furthermore, semimetal-based ceramic buffers, such as Si- and Ge-based layers, have been shown to significantly influence interfacial properties and hysteresis behavior by suppressing interfacial reactions and controlling oxidation conditions [98, 99]. Ko et al. systematically compared buffer layers composed of Si, Ge, Hf, La, and Gd on monolayer MoS_2_ and, via XPS analysis, demonstrated that Si and Ge form stable interfaces for ALD HfO_2_ deposition without inducing significant damage to MoS_2_ (Fig. 6c) [98]. However, the oxidation conditions of Si-based buffers critically affect the defect structure and fixed-charge distribution of the resulting SiO_x_ layers, thereby modifying interfacial interactions with the 2D channel [99]. Under insufficient oxidation conditions (oxygen gas pressure ≈ 0.2 Pa), excessive oxygen vacancies and under-coordinated Si^n+^ defect centers are formed, which act as electron trap states and induce electrical instability (Fig. 6d). As a result, the energy bands near the MoS_2_ channel shift upward, effectively increasing the Schottky barrier height and leading to a positive threshold-voltage shift.

To address the limitations of three-dimensional network ceramics—such as vacancy formation and dangling-bond-induced trap states—molecular-crystal ceramics, such as Sb_2_O_3_, have recently attracted attention as buffer layers that form vdW interfaces with 2D materials and provide near-trap-free environments [100]. Xu et al. [101] employed a thermally evaporated Sb_2_O_3_ buffer layer to achieve uniform HfO_2_ growth on MoS_2_, enabling the fabrication of dual-gate FETs. Raman spectra confirm that the characteristic peaks of monolayer MoS_2_ are preserved even after the deposition of Sb_2_O_3_ and HfO_2_, indicating negligible structural damage (Fig. 6e). The resulting devices achieved SS values as low as 60 mV·dec^−1^ (Fig. 6f), with an extracted interface trap density of ~ 1.4 × 10^11^ cm^−2^·eV^−1^ for a total dielectric thickness of 5 nm (Sb_2_O_3_: 1 nm, HfO_2_: 4 nm).

In addition, 2D or atomic-layer-based buffers, such as GrO and amorphous carbon monolayers (ACM), have been reported to be effective in providing nucleation sites and suppressing interfacial diffusion [66, 102]. Wyndaele et al. introduced GrO, formed through cleaning (H_2_ DPS, 3 s) and UV–O_3_ oxidation (30 min), as a buffer layer to induce uniform HfO_2_ growth on monolayer WS_2_ [66]. The incorporation of the GrO buffer resulted in an areal density comparable to that on SiO_2_ substrates, and cross-sectional scanning TEM (STEM) analysis confirmed the formation of uniform and continuous HfO_2_ layers in the buffered structure (Fig. 6g, h).

Overall, buffer/dielectric bilayer architectures enhance dielectric nucleation and uniformity, enable passivation during potentially damaging processes such as PEALD, and extend applicability to oxidation- or moisture-sensitive materials as well as synaptic device implementations [103–108]. At the same time, several trade-offs must be considered, including structural damage induced by high-energy metal deposition [109], electrical instability arising from defect-rich buffer layers [110], and an inevitable reduction in effective dielectric constant due to the introduction of an additional interlayer [89]. The electrical characteristics of ALD-based devices employing various buffer layers are systematically compared in Table 1. Consequently, careful optimization of buffer-layer thickness and material selection is required to maximize the benefits of buffer-layer strategies.

**Table 1:** Summary of dielectric properties and device performance of state-of-the-art dielectrics integrated on 2D semiconductors

Type (TMDs)	Buffer layer (methods)	EOT [nm]	RMS roughness [nm]	I_on_/I_off_	SS [mV/dec]	References
n (MoS_2_)	AlO_x_ (Al ~ 2 nm, EBE → air oxidation AlO_x_)	15.68(TG)*	0.93	≥ 10^7^	930	[69]
n (MoS_2_)	AlO_x_ (Al ~ 1 nm, EBE → air oxidation AlO_x_)	7.3(TG)*	NA	2.5 × 10^6^	NA	[70]
p → n (WSe_2_)	AlO_x_ (Al ~ 1 nm, EBE → air oxidation AlO_x_)	1.21(TG)*	NA	≥ 10^7^	NA	[71]
n (MoS_2_)	Al_2_O_3_ (Al_2_O_3_ ~ 1 nm, EBD)	4.1(TG)*	0.50	≥ 4 × 10^8^	103	[78]
n (MoS_2_)	AlO_x_ (Al ~ 1 nm, EBE → air oxidation AlO_x_)	7.8(TP)*	NA	~ 10^6^	NA	[70]
Ambi → n (WS_2_)	AlO_x_ (Al ~ 1.5 nm, EBE → air oxidation AlO_x_ ~ 4 nm)	1.80(DG)*	0.28	10^10^	88	[77]
n (MoS_2_)	Al_2_O_3_ (AlO_x_, ALD 10 cycles, 80 °C, short purge 5 s)	4.78(TG)*	0.78	≥ 10^5^	≥ 88	[79]
n (MoS_2_)	AlN (AlN ~ 2 nm, PEALD, 170 °C)	8.67(TG)**	0.54	10^6^	NA	[81]
n (MoS_2_)	AlO_x_ (AlO_x_ ~ 5 nm, ALD, 100 °C)	285(BG)* 6.34(TG)*	NA	2.5 × 10^2^	NA	[83]
n (MoS_2_)	Al_2_O_3_ (UV-ozone, RT, 15 min → Al_2_O_3_ ~ 3 nm, ALD. 200 °C)	2.03(DG)*	NA	≥ 10^4^	~ 60	[84]
n (MoS_2_)	AlO_x_ (AlO_x_ ~ 2 nm, Nanofog, 50 °C)	1.33(TG)	NA	≥ 10^6^	100	[86]
n (MoS_2_)	Y_2_O_3_ (Y ~ 1 nm, TE → air oxidation Y_2_O_3_ ~ 1 nm)	6.84(DG)*	NA	10^6^	115	[88]
n (MoS_2_)	HfO_x_ (Hf ~ 3 nm, EBE → air exposure HfO_x_ ~ 3.4 nm)	0.81(TG)*	0.22	10^6^	180	[89]
n (MoS_2_)	TiO_x_ (Ti ~ 1.2 nm, EBE → air oxidation)	2.36(BG)**	NA	2.7 × 10^8^	64	[91]
n (MoS_2_)	TaO_x_ (Ta ~ 1 nm, EBE → air oxidation TaO_x_ ~ 2.5 nm)	0.95(DG)	NA	> 10^5^	~ 70	[95]
n (MoS_2_)	Y_2_O_3_ (Y ~ 2 nm, EBE → O_2_ furnace oxidation)	1.85(DG)**	NA	≥ 10^8^	~ 60	[96]
n (MoS_2_)	SiO_x_ (Si ~ 0.4 nm, EBE → air oxidation)	0.99 ± 0.1(DG)	NA	10^8^	~ 80	[98]
n (MoS_2_)	SiO_x_ (Si ~ 0.2 nm, EBE → air exposure)	0.9(TG)	NA	≥ 10^7^	~ 70	[111]
n (MoS_2_)	SiO_x_ (SiO_x_ ~ 2 nm, EBE → O_2_ annealing 100 °C, 60 min, 2 Pa)	4.73(DG)*	NA	≥ 10^8^	≥ 114	[99]
n (MoS_2_)	NA (only Sb_2_O_3_, TE)	~ 6.7(DG)*	NA	10^7^	68	[100]
n (MoS_2_)	Sb_2_O_3_ (Sb_2_O_3_ ~ 1 nm, TE)	1.01(TG)	NA	5 × 10^7^	> 60	[101]
n (MoS_2_)	Al_2_O_3_ (Al_2_O_3_ ~ 5 nm, ALD, 150 °C)	11.1(TG)**	NA	10^6^	NA	[108]
n (MoS_2_)	Al_2_O_3_ (UV-ozone RT. 15 min → Al_2_O_3_ ~ 3 nm, ALD 200 °C)	2.03(DG)*	NA	10^6^	~ 60	[85]

(*: EOT values were directly calculated in this work using κ ≈ 25 for HfO_2_ and ZrO_2_, κ ≈ 8 for Al_2_O_3_, κ ≈ 14 for Y_2_O_3_, and κ ≈ 11.5 for Sb_2_O_3_, **: Cases in which only the dielectric constant was reported in the original literature.)

#### Organic layer

**Fig. 7:**
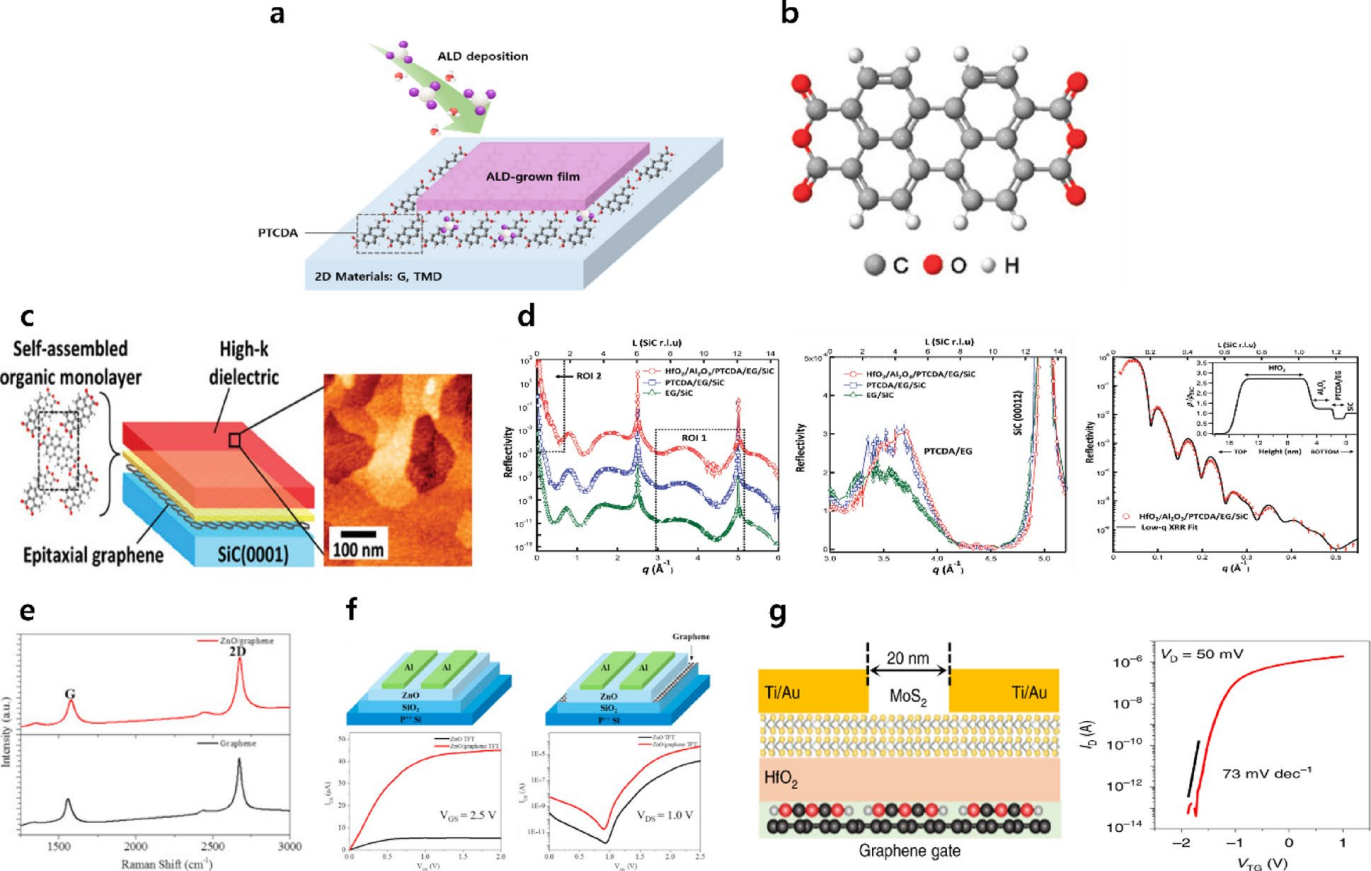
**a** Schematic illustration of the hybrid PTCDA/HfO_2_ gate stack on 2D materials. **b** Molecular structure of PTCDA. **c** High-κ dielectric grown on epitaxial graphene enabled by a self-assembled PTCDA monolayer, with AFM images acquired immediately after PTCDA deposition and subsequent ALD of Al_2_O_3_ [115]. **d** X-ray reflectivity analysis of ALD dielectric films and the underlying PTCDA/epitaxial graphene interface [115]. **e** Raman spectra of ZnO/graphene thin film and PTCA-treated graphene [118]. **f** Device schematics, output characteristics, and transfer characteristics of ZnO and ZnO/graphene TFTs [118]. **g** Device schematic and transfer characteristics of a short-channel MoS_2_ FET with a PTCDA/HfO_2_ gate stack (*L*_g_ = 20 nm) [13]

Organic buffer layers have also been proposed as an alternative strategy to avoid channel damage that can arise during metal deposition in metal-oxide-based buffer schemes. Certain organic materials act as hydrophilic coatings on 2D surfaces, thereby promoting the adsorption of ALD precursors and facilitating uniform nucleation **(**Fig. 7a). Representative organic buffer layers include 3,4,9,10-perylenetetracarboxylic dianhydride (PTCDA), perylene tetracarboxylic acid (PTCA), polypropylene carbonate (PPC), polymethylmetacrylate (PMMA), titanyl phthalocyanine (TiOPc), and various self-assembled monolayers (SAMs) [13, 112–123]. In the case of PTCDA, the molecular structure features a perylene core that enables ordered alignment on graphene via π–π interactions (Fig. 7b) [13, 115]. In addition, oxygen-containing terminal groups stabilize the monolayer through strong noncovalent interactions and provide chemical functionality [119]. Alaboson et al. fabricated metal–oxide–graphene capacitors incorporating a PTCDA buffer layer with a dielectric stack of 3 nm Al_2_O_3_ and 10 nm HfO_2_ (Fig. 7c) [115]. X-ray reflectivity (XRR) analysis confirmed that the buried PTCDA/graphene interface remained structurally intact after ALD. At the same time, a smooth and uniform HfO_2_/Al_2_O_3_ dielectric stack was successfully formed atop the PTCDA/graphene layer (Fig. 7d). The devices exhibited a high capacitance of approximately 700 nF·cm^−2^ and a low leakage current of ~ 5 × 10^−9^ A·cm^−2^ at 1 V bias, outperforming previously reported top-gated graphene FETs in terms of both capacitance and leakage. This work demonstrated that uniform dielectric deposition can be achieved on graphene without inducing damage, establishing an effective route toward high-performance nanoscale devices.

Wirtz et al. reported stable dielectric deposition on TMDs using perylene bisimide as a buffer layer [119]. The perylene molecules possess a large aromatic core with hydroxyl (–OH) and carboxyl (–COOH) terminal groups, enabling noncovalent attachment to TMD surfaces. Compared to PTCDA, these molecules feature longer terminal chains containing carboxylic acid groups. AFM analysis revealed that perylene adsorption is sparse on SiO_2_ but preferentially forms vertically aligned structures on TMD surfaces. Raman spectroscopy confirmed negligible peak changes before and after dielectric deposition (Fig. 7e) [118], indicating that the TMD lattice remains intact. A slight blue shift suggested weak p-type doping. These results confirmed that Al_2_O_3_ can be deposited on TMDs in a nondestructive, stable manner using perylene bisimide buffer layers.

Liu et al. demonstrated successful dielectric deposition using PTCA as a buffer layer, enabling the fabrication of ZnO and ZnO/graphene thin-film transistors (TFTs) with high on/off ratios (Fig. 7f) [118]. SEM and Raman analyses confirmed that PTCA treatment does not perturb the graphene lattice. Owing to its conjugated ring structure and terminal carboxylate (–COO^−^) groups, PTCA serves as an effective functionalization layer. AFM measurements showed uniform ZnO film formation on PTCA-treated graphene. The resulting ZnO and ZnO/graphene TFTs exhibited mobilities of μ ≈ 7.35 and 18.21 cm^2^·V^−1^·s^−1^, respectively, with on/off ratios of 1.53 × 10^6^ and 1.68 × 10^7^, indicating a twofold mobility enhancement and superior switching behavior. The surface –OH species on PTCA were found to react efficiently with ALD precursors, enabling uniform ZnO growth.

More recently, Li et al. [13] utilized PTCDA as a self-limited, vdW–bonded monolayer buffer formed via an epitaxial process on 2D materials (Fig. 7g). AFM and STEM analyses of HfO_2_/monolayer PTCDA/2D structures revealed an additional thickness of only 0.3–0.4 nm and an ultralow roughness (*R*_q_ ≈ 0.13 nm). In contrast, direct deposition without PTCDA led to highly nonuniform growth. Uniform HfO_2_ films as thin as 2 nm were achieved over micrometer-scale device areas on graphene, hBN, and TMDs (MoS_2_, WS_2_, MoSe_2_, and WSe_2_). Collectively, perylene-based organic buffer layers provide an effective, nondestructive strategy for dielectric integration with 2D materials, enabling uniform, ultrathin oxide growth while preserving the intrinsic properties of the underlying channels.

### Process innovation: ALD modification

Conventional thermal ALD has been widely used for depositing high-κ thin films; however, it suffers from several inherent limitations. Due to the limited surface reactivity of certain precursors and reactants, nucleation can be delayed, leading to incomplete or nonuniform film growth. In addition, relatively high process temperatures are often required. These issues become particularly critical when thermal ALD is applied to atomically thin 2D materials that lack dangling bonds, where uniform dielectric films are difficult to achieve, and channel damage may occur, ultimately degrading device performance. To overcome these limitations, various modified ALD processes have been proposed. Representative approaches include PEALD, O_3_-based ALD, and precursor engineering strategies. In this section, we review recent studies that address the drawbacks of conventional thermal ALD by employing these alternative techniques to enable high-quality thin-film deposition on 2D materials.

#### PEALD

PEALD is an alternative ALD technique capable of depositing high-quality dielectric layers [124]. In PEALD, plasma is employed during the deposition process to activate surface reactions [125]. Owing to this characteristic, thin-film growth can be achieved at relatively low temperatures, enabling the formation of high-quality insulating layers without relying on substrate heating, in contrast to conventional thermal ALD [60]. In addition, plasma species are more readily removed than the reactant gases used in thermal ALD, leading to shorter purge times. Consequently, PEALD offers both lower-temperature processing and higher deposition throughput compared to thermal ALD [126].

Walter et al. employed PEALD to deposit ZnO thin films on MoS_2_ and WSe_2_ surfaces [65]. Because plasma is applied concurrently with deposition, ZnO growth occurred after only a short nucleation delay on 2D TMDs. However, oxidation of the upper layers of the TMDs was observed following plasma exposure. This behavior contrasts with thermal ALD processes incorporating UV–O_3_ pretreatment. In the case of PEALD-grown ZnO on MoS_2_ and WSe_2_, uniform and continuous films were formed across the entire surface, whereas UV–O_3_ pretreatment led to more limited, material-dependent growth. This difference arises from distinct surface-activation mechanisms. UV–O_3_ treatment primarily induces the adsorption of oxygen atoms, thereby generating a limited density of reactive sites. In contrast, plasma exposure directly oxidizes the surface, creating a higher density of reactive sites. As a result, PEALD enables more uniform thin-film deposition on TMD surfaces.

#### Ozone-based reactant

Ozone reactants exhibit high reactivity and volatility, enabling shorter purge times between ALD cycles compared to other oxidizing reactants [127]. Cheng et al. employed ozone-based ALD to deposit Al_2_O_3_ on MoS_2_ and systematically analyzed the interfacial properties [64]. In this study, the TMA + H_2_O process was compared with the TMA + O_3_ process. When O_3_ was used as the reactant, a more uniform Al_2_O_3_ film with a thickness of 3.1 nm and an RMS roughness of 1.2 nm was obtained, in contrast to the H_2_O-based process (Fig. 5k). This behavior was attributed to the decomposition of ozone into oxygen molecules and highly reactive atomic oxygen upon reaching the surface, which promotes nucleation of the TMA precursor. To further improve film uniformity, an initial seed layer was formed by performing five cycles of the TMA + O_3_ reaction, followed by additional deposition at 200 °C. As a result, the RMS roughness was reduced to 0.23 nm, and a uniform Al_2_O_3_ film was achieved without inducing oxidation of the MoS_2_ surface. These results indicate that using ozone as a reactant enables more uniform thin-film growth on TMD surfaces. In contrast, Park et al. investigated the passivation performance of Hf_1-x_Zr_x_O_2_ (HZO) thin films deposited on WS_2_ as a function of ozone concentration. When O_3_-based HZO ALD was performed at 260 °C, monolayer WS_2_ underwent severe oxidation, becoming optically transparent and exhibiting significant structural degradation [128]. However, under identical conditions using H_2_O as the reactant, no meaningful changes were observed in the Raman and photoluminescence (PL) spectra of WS_2_. Cross-sectional TEM analysis further confirmed that monolayer WS_2_ was oxidized after O_3_-based ALD, whereas the WS_2_ layer remained intact after H_2_O-based ALD.

Taken together, these two studies demonstrate that, even when the same O_3_ reactant is used, the presence or absence of a seed layer decisively determines channel oxidation. This highlights that not only the choice of precursor and reactant, but also the detailed process sequence and device structure, critically influence the final thin-film properties in ALD processes. Based on these considerations, a systematic comparison of the processing conditions and outcomes associated with thermal ALD, surface modification, PEALD, and O_3_-based ALD is necessary. Accordingly, the detailed conditions and key characteristics of the ALD processes reported to date are summarized in Table 2.

**Table 2:** Comparative summary of process conditions and characteristics for ALD pretreatments and precursors

2D material	ALD material	Treatment	Precursor	Reactant	Temperature (℃)	Surface morphology	RMS value (nm)	References
Graphene	Al_2_O_3_	NO_2_	TMA	NO_2_	25	–	–	[53]
HOPG	Al_2_O_3_	NO_2_/TMA	TMA	H_2_O	150	film	–	[55]
MoS_2_	Al_2_O_3_	O_2_ plasma	TMA	H_2_O	250	film	0.07	[60]
	HfO_2_	O_2_ plasma	TEMAHf	H_2_O	250	film	0.18	[60]
	Al_2_O_3_	O_2_ plasma	TMA	H_2_O	200	film	0.58	[52]
		H_2_O plasma	TMA	H_2_O	200–250	film	0.16	[63]
	Al_2_O_3_	–	TMA	O_3_	200	film	0.23	[64]
		–	TMA	H_2_O	200	island	–	[64]
	ZnO	UV-O_3_	DEZ	H_2_O	125	film	0.55	[65]
		PEALD	DEZ	N_2_O	125	film	≤ 0.2	[65]
WSe_2_	ZnO	UV-O_3_	DEZ	H_2_O	125	island	0.55	[65]
		PEALD	DEZ	N_2_O	125	film	≤ 0.2	[65]
WS_2_	HfO_2_	Gr + UV-O_3_	Hf chloride	H_2_O	300	film	1–1.5	[66]
	HZO	–	TEMAHf TEMAZr	O_3_	260	film	–	[128]
		–	TEMAHf TEMAZr	H_2_O	260	film	–	[128]

#### Substrate-driven ALD

Activation-based approaches such as plasma treatment, UV–O_3_ exposure, and O_3_-assisted ALD can enhance the surface reactivity of 2D materials. However, they inherently entail trade-offs, including increased defect density and degradation of carrier mobility [60, 64, 129–135]. Similarly, seed-layer or buffer-layer strategies employing materials such as Al, PTCDA, and organic SAMs improve dielectric nucleation but often degrade performance due to increased interface trap density and enhanced charge scattering [136]. To overcome these limitations, a substrate-assisted approach has been proposed, in which the surface reactivity of 2D materials is modulated by exploiting the physical and electronic properties of the underlying substrate, without introducing any chemical treatment to the channel itself. Dlubak et al. compared the ALD growth of Al_2_O_3_ (10 nm) on monolayer graphene supported on Cu and SiO_2_ substrates and, via SEM analysis, observed defect-free, uniform dielectric coverage exclusively on Cu-supported graphene [137]. Notably, the same level of uniformity was maintained even for ultrathin 3-nm Al_2_O_3_ films. This substrate-dependent behavior was attributed to the electronic transparency of monolayer graphene to external electric fields, whereby polar traps formed at the graphene–metal interface due to the work function mismatch penetrate through the graphene layer and attract ALD precursors, thereby increasing the density of nucleation sites [138].

It has been reported that the strain state, charge-transfer characteristics, and dielectric screening of 2D semiconductors vary depending on the underlying metallic substrate, leading to a redistribution of their electronic structure [139]. Such substrate-induced electronic perturbations enable uniform dielectric nucleation without chemical functionalization of the 2D surface, positioning substrate-assisted ALD as a seed-free and nondestructive growth strategy. Bayer et al. directly visualized this effect using STEM, showing that the nucleation coverage of HfO_2_ on monolayer graphene supported by Cu reached approximately 54%, whereas it dropped to ~ 20% for bilayer graphene [140]. This observation clearly demonstrates that the electronic properties of the underlying Cu catalyst actively participate in the initial ALD reactions through field penetration. Despite these advantages, substrate-assisted ALD strategies still face limitations related to electrostatic screening effects, as the penetration of substrate-induced electric fields is significantly reduced in multilayer 2D materials, thereby weakening the substrate influence. However, since this effect is significant only in monolayer systems, further investigations are required to address this limitation.

### vdW transfer and dry integration

**Fig. 8:**
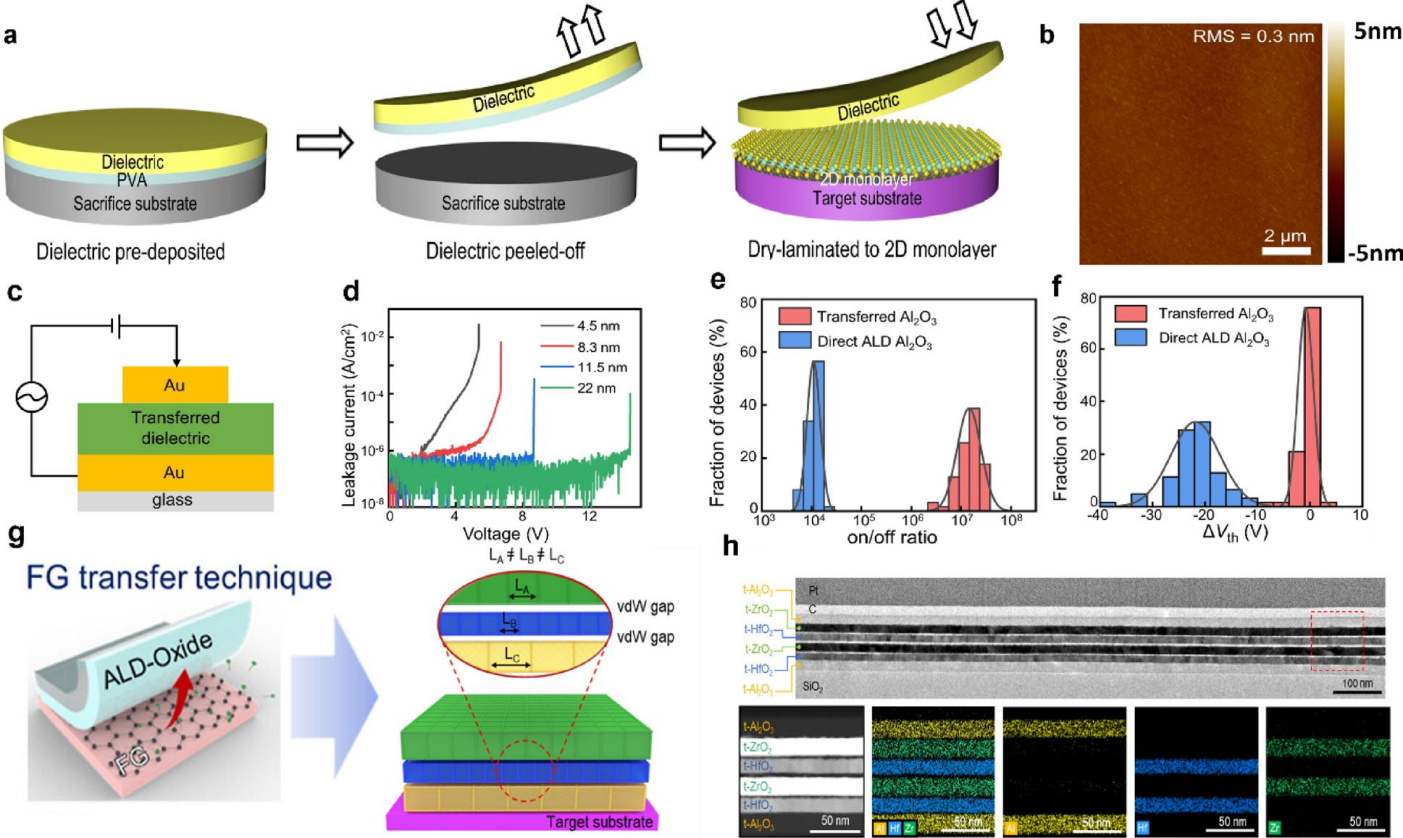
**a** Schematics of wafer-scale dielectric lamination process with three steps [19]**. b** AFM height measurement for the bottom side of transferred Al_2_O_3_ [19]. **c** Schematic of MIM device structure for leakage current measurement [19]. **d** Leakage current of transferred Al_2_O_3_ with various thicknesses. The statistical distribution of **e** on/off ratio and **f** threshold voltage shift of top-gated MoS_2_ FETs with transferred Al_2_O_3_ and direct ALD Al_2_O_3_, respectively [19]. **g** Schematic illustration of FG-based transfer of ALD-oxide and the transferred multilayer dielectric stacks with various oxide layers [148]. **h** Cross-section TEM image and EDS mapping image of Al_2_O_3_/ZrO_2_/HfO_2_/ZrO_2_/HfO_2_/Al_2_O_3_, multilayer dielectric stack [148]

Conventional ALD-based direct deposition on 2D materials inherently suffers from degraded film quality because pristine surfaces lack dangling bonds. Although surface pretreatments and buffer layers have been introduced to enhance nucleation, these approaches often induce defect formation, interfacial trap states, and degraded gate controllability, thereby limiting device performance and reliability [131, 141–143]. As an alternative to direct deposition and chemically assisted approaches, vdW lamination enables dielectric integration through dry transfer, thereby decoupling dielectric quality from surface reactivity constraints of 2D materials [19, 144–149]. Because the interface between the dielectric and the 2D material in vdW lamination is governed by vdW interactions, this approach effectively circumvents the issues commonly encountered during direct deposition. Zheyi Lu et al. proposed a wafer-scale dry transfer strategy in which ultrathin high-κ dielectrics, including Al_2_O_3_ and HfO_2_, were pre-deposited by ALD on an ultrathin poly(vinyl alcohol) sacrificial layer and subsequently transferred onto monolayer MoS_2_ [19]. During the dry lamination process, uniform stacking was achieved across the entire wafer, without forming cracks or wrinkles. AFM analysis revealed that the transferred dielectrics maintained atomically smooth surfaces with an RMS roughness of approximately 0.3 nm (Fig. 8a, b**)**. Electrical characterization of metal–insulator–metal (MIM) structures fabricated using the transferred dielectrics demonstrated leakage current densities below 10^–7^ A·cm^−2^ up to breakdown over a thickness range of 4.5–22 nm, significantly surpassing the International Technology Roadmap for Semiconductors (ITRS) requirement of 10^−2^ A·cm^−2^ **(**Fig. 8c, d**)**. Moreover, aggressive scaling to an EOT of 1.2 nm was achieved without damaging the MoS_2_ channel. Top-gated MoS_2_ FETs fabricated using the transferred dielectrics exhibited a markedly reduced threshold-voltage shift (~ 0.8 V) compared to devices with directly deposited ALD Al_2_O_3_, while preserving the intrinsic on/off current ratio **(**Fig. 8e, f**)**. Notably, n-type doping, trap generation, hysteresis, and threshold instability were largely suppressed. These results demonstrate that transfer-based high-κ dielectrics enable large-area integration of ultrathin dielectric layers without compromising the intrinsic properties of 2D semiconductors.

Venkatakrishnarao et al. introduced a distinct vdW lamination strategy employing a liquid–metal oxide as a sacrificial layer to transfer high-quality ALD-grown HfO_2_ onto 2D materials [147]. In this approach, an ultrathin Ga_2_O_3_ layer (≈3 nm), formed via spontaneous oxidation on liquid Ga, provides a hydrophilic surface with dangling bonds that stabilize ALD nucleation. The Ga_2_O_3_ layer remains robust under thermal, plasma, and UV exposure, enabling uniform HfO_2_ growth even at high temperatures during ALD. The transferred HfO_2_ films (RMS roughness ≈ 0.4 nm) exhibited crack-free and wrinkle-free lamination over large areas, and atomically smooth interfaces without step-height variations were confirmed for both monolayer and multilayer WS_2_. Furthermore, by simultaneously transferring HfO_2_ and Au electrodes in a single-step vdW stack integration process, this approach demonstrated strong potential for backend-of-line (BEOL) compatibility and multilayer circuit integration.

More recently, Kim et al. proposed a fluorinated graphene (FG)-based transfer technique that fundamentally eliminates lattice mismatch, intermixing, and thermal degradation issues associated with conventional direct ALD processes, thereby enabling upper-tier device fabrication in monolithic three-dimensional (M3D) architectures [148]. The C–F dipoles on the FG surface stabilize ALD nucleation, allowing uniform growth of various dielectric oxides. Subsequent annealing at 400 °C cleaves the C–F bonds, enabling clean, undamaged delamination of the dielectric films. Even in transferred multilayer dielectric stacks such as Al_2_O_3_/HfO_2_/ZrO_2_, the presence of a vdW gap prevents strain accumulation and overcomes the limitations of lattice mismatch **(**Fig. 8g, h**)**. Importantly, no interfacial diffusion was observed after post-annealing at 400 °C, addressing critical challenges related to strain and intermixing in M3D integration. These characteristics indicate that transfer-based dielectric oxides can maintain high dielectric performance under BEOL-compatible low-thermal-budget processes while satisfying the stringent electrical reliability requirements of M3D integration.

### Post-treatment

**Fig. 9:**
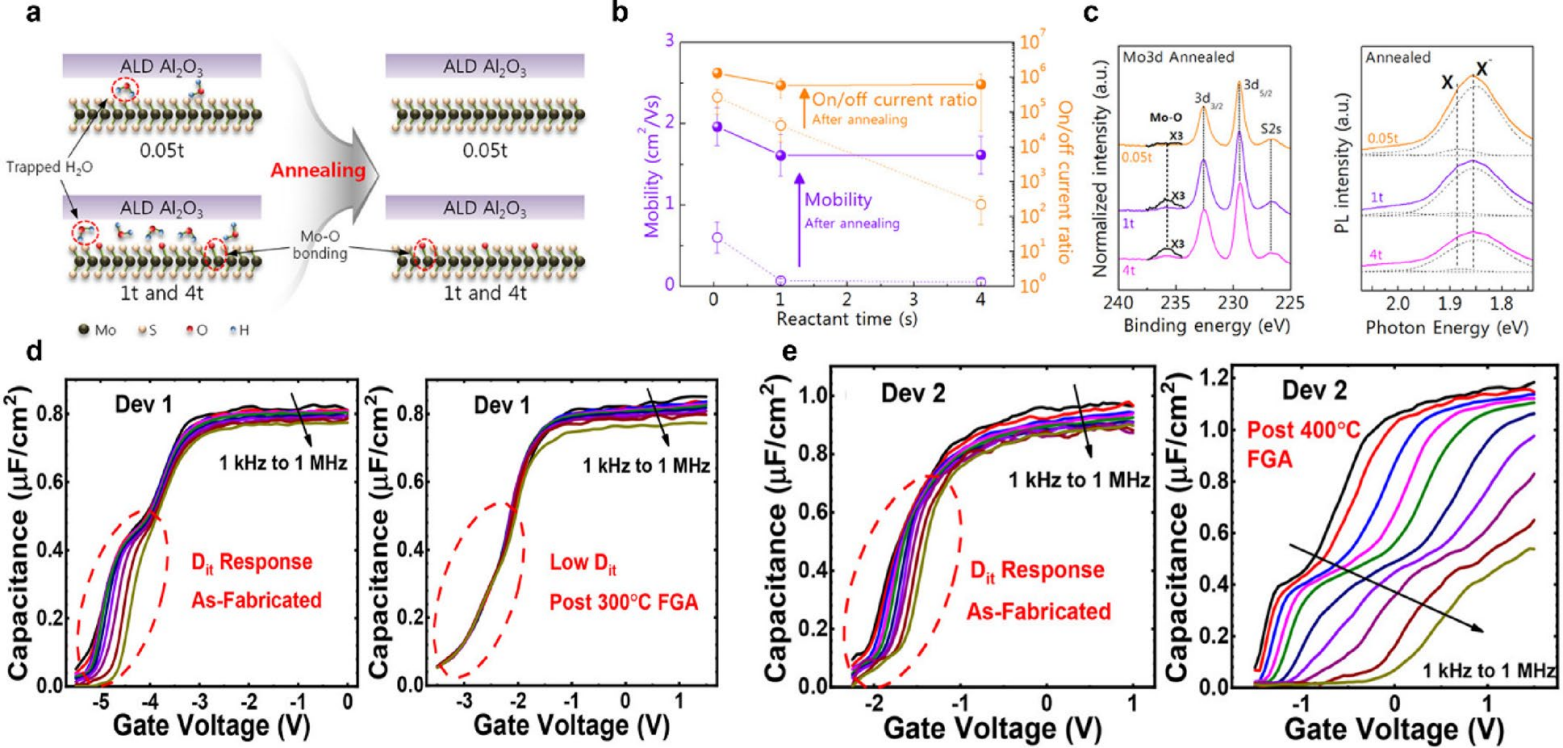
**a** Schematic illustration of ALD grown Al_2_O_3_ on 1L MoS_2_ before and after annealing [155]. **b** Extracted field effect mobility and on/off ratio of top-gate 1L MoS_2_ FETs before and after annealing [155]. **c** XPS spectra of the Mo 3d core levels and corresponding PL spectra measured after annealing to identify the presence of Mo-based oxides in samples processed under different H_2_O reactant exposure times [155]. *C* − *V* characterization of HfO_2_/Al_2_O_3_/ MoS_2_ gate stack (1 kHz to 1 MHz) before/after forming gas annealing **d** at 300 °C and **e** at 400 °C. Large frequency dispersion indicates a significantly higher *D*_it_ due to the annealing [154]

ALD on 2D semiconductors often suffers from limited initial nucleation due to their intrinsically low surface reactivity, leading to non-uniform, island-like growth. Such morphological nonuniformity can be partially mitigated by subsequent thermal annealing. Immediately after deposition, ALD films typically remain in an amorphous state, and residual species such as –OH and –CH_3_ ligands, as well as oxygen vacancies, are commonly incorporated into the film [150–154]. These residual defects act as charge traps, giving rise to increased leakage current, pronounced hysteresis, and degraded SS. Song et al. investigated the effects of ALD process parameters and post-deposition annealing on top-gated monolayer MoS_2_ transistors employing a 40 nm-thick ALD Al_2_O_3_ gate dielectric [155]. By systematically varying the H_2_O reactant exposure time during ALD, they observed that increased H_2_O exposure led to the formation of Mo–O bonds on the MoS_2_ surface and to the accumulation of H_2_O molecular traps at the Al_2_O_3_/MoS_2_ interface. These effects resulted in a pronounced degradation of carrier mobility and the *I*_on_/*I*_off_ ratio. Subsequent post-deposition annealing at 120 °C for 24 h under ultra-high-vacuum conditions effectively removed interfacial H_2_O molecules, leading to an improvement in mobility from 0.6 to 2.0 cm^2^·V^−1^·s^−1^ and an enhancement of *I*_on_/*I*_off_ by more than three orders of magnitude (Fig. 9a, b). XPS and PL analyses revealed that while annealing could not eliminate Mo–O bonding, it played a critical role in suppressing charge scattering originating from interfacial moisture (Fig. 9c).

Wen et al. examined the influence of annealing ambient on MoS_2_ transistors incorporating an ALD HfTiO gate dielectric [156]. Annealing was performed at 400 °C for 10 min under N_2_, O_2_, and NH_3_ atmospheres, and the NH_3_-annealed devices showed the greatest performance enhancement. In particular, the field-effect mobility increased by approximately 3.4 times, from 9.2 to 31.1 cm^2^·V^−1^·s^−1^, while SS was reduced from 363 to 100 mV·dec^−1^. These results indicate that the chemical composition of the annealing ambient directly affects interfacial defect density and charge-scattering mechanisms. Zhao et al. analyzed annealing effects in top-gated few-layer MoS_2_ transistors with an 8 nm-thick ALD HfO_2_ gate dielectric [157]. When annealed at 300 °C under N_2_ or forming gas conditions, an increase in drain current was observed. However, incomplete channel depletion and a negative shift in threshold voltage were also noted. In contrast, forming-gas annealing at 400 °C enabled deeper hydrogen diffusion into the interface, effectively passivating oxygen vacancies and interfacial defects. As a result, the threshold voltage decreased by approximately 3 V, accompanied by simultaneous improvements in hysteresis and gate leakage. Nevertheless, a trade-off was identified. A slight reduction in drain current was observed due to thermal degradation of the contact or the MoS_2_ channel.

In a subsequent study, Zhao et al. [154] investigated the interfacial defect characteristics of top-gated few-layer MoS_2_ transistors employing an HfO_2_ (6 nm)/Al_2_O_3_ (3 nm) gate stack through *C*–*V* analysis after forming-gas (N_2_/H_2_) annealing at 300 °C and 400 °C. After annealing at 300 °C, hydrogen-induced passivation nearly eliminated interface traps, resulting in the disappearance of frequency dispersion in the *C*–*V* curves and a reduction of *D*_it_ to below the measurement limit. Concurrently, the positive fixed charge density within the dielectric decreased from 5.1 × 10^13^ to 2.6 × 10^13^ cm^−2^. A small degree of unintentional n-type doping was activated in MoS_2_ (Fig. 9d). In contrast, devices annealed at 400 °C exhibited a pronounced increase in *C*–*V* dispersion, with *D*_it_ rising sharply to ~ 8.3 × 10^13^ cm^−2^·eV^−1^, indicating severe interfacial degradation. These results demonstrate that the Al_2_O_3_/MoS_2_ interface is highly sensitive to high-temperature annealing and that excessive thermal energy can induce the formation of new interfacial defects (Fig. 9e).

Collectively, these studies highlight that while post-deposition annealing can effectively mitigate moisture-related traps and passivate interfacial defects in 2D-material-based FETs, high-temperature processing often introduces competing degradation mechanisms. Therefore, low-temperature annealing strategies that carefully control ambient chemistry and process pressure are required to balance defect passivation and thermal stability in 2D semiconductor devices.

### Comprehensive evaluation

**Fig. 10:**
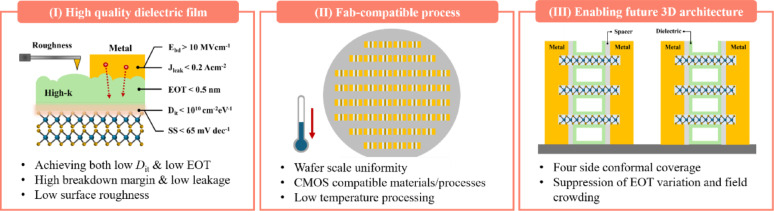
Requirements for dielectric integration in 2D materials-based electronics

To date, a range of strategies for achieving reliable dielectric deposition on 2D materials has been explored. However, as discussed throughout the preceding sections, each approach exhibits distinct advantages and inherent limitations. Accordingly, rather than evaluating these strategies in isolation, a systematic comparison is required to identify the conditions under which each approach becomes advantageous or constrained. From this perspective, Fig. 10 outlines three key criteria for assessing dielectric integration strategies in 2D TMD-based devices. The significance of each requirement is summarized as follows:

(I) *High-quality dielectric film*: Beyond achieving uniform coverage, it is essential to simultaneously meet the requirements of EOT scaling and superior interfacial properties. According to the International Roadmap for Devices and Systems (IRDS) 2031 targets, the relevant performance metrics include breakdown field > 10 MV cm^−1^, *J*_leak_ < 0.2 A cm^−2^, EOT < 0.5 nm, *D*_it_ < 10^10^ cm^−2^ eV^−1^, and SS < 65 mV dec^−1^ [158].
(II) *Fab-compatible process*: For practical implementation in semiconductor manufacturing environments, dielectric integration must rely on CMOS-compatible materials and processes. This includes achieving conformal dielectric deposition over wafer-scale substrates while maintaining compatibility with low-temperature processing constraints
(III) *Enabling future 3D architecture*: For next-generation 3D device configurations incorporating 2D semiconductors, dielectric integration must ensure conformal coverage on all sides while minimizing EOT variation and mitigating electric-field crowding in densely stacked geometries. In addition to planar device considerations, this requires maintaining dielectric isolation, thermal stability, and device reliability in vertically integrated structures.

Overall, dielectric integration strategies for 2D TMDs can be evaluated along three primary axes: film quality, manufacturability, and structural scalability. Table 3 provides a comparative overview of the representative approaches discussed above, highlighting their relative strengths and key characteristics with respect to these requirements. This comparison indicates that no single approach currently satisfies all three requirements simultaneously; rather, each strategy involves inherent trade-offs. Consequently, future efforts toward dielectric integration in next-generation 2D electronic devices should focus on minimizing these trade-offs while balancing performance and process compatibility.

**Table 3:** Evaluation of dielectric integration strategies for 2D materials with respect to key requirements defined in Fig. 10

Methods	Category		Requirements			Features	References.
			(Ⅰ)	(Ⅱ)	(Ⅲ)		
Surface modification	UV-ozone & plasma treatment		●	●●◐	●◐	Process simplicity Interfacial damage	[60, 62, 64, 159, 160]
Buffer layer deposition	Non- vdW buffer	Organics	●◐	●●	●◐	Process simplicity Limited thermal stability, Interfacial damage	[113]
		Inorganics	●●	●●●	●●	High process compatibility Interfacial damage	[77, 79, 86]
	vdW buffer	Organics	●●◐	●◐	●◐	High-quality interface Limited thermal stability	[115, 119]
		Inorganics	●●●	●◐	●◐	High-quality interface Limited material selection	[66, 100, 101]
Process innovation	Substrate-driven ALD		–	●◐	●	Modification-free Dependence on substrate & thickness	[137, 138]
	VdW transfer		●●◐	●	●◐	High-quality interface Low process compatibility	[138, 145–148]

## 2D/3D gate stacks for 2D FETs: interface physics and dielectric materials

### 2D/3D interfaces in 2D FET gate stacks

The gate stack in 2D FETs typically combines 2D semiconductors such as MoS_2_, WSe_2_, WS_2_, and MoSe_2_ with 3D dielectrics including SiO_2_, Al_2_O_3_, HfO_2_, ZrO_2_, and layered oxides [3, 42]. In 2D devices, hBN is used as an interfacial layer or for full encapsulation. The absence of dangling bonds on TMD and hBN basal planes means that the Si/SiO_2_ (dense covalent bonds) is replaced by a spectrum of interfaces from vdW to partially covalent and defect-mediated interfaces. This governs nucleation, trap formation, band alignment, remote doping, and carrier scattering [4, 161]. Recent roadmaps and perspectives on 2D electronics further highlight that achieving low-defect, high-κ gate stacks with atomically clean 2D/dielectric interfaces has become one of the primary bottlenecks for scaling 2D FETs toward sub-3-nm technology nodes [2, 162, 163].

#### vdW interface and partially covalent interface

**Fig. 11:**
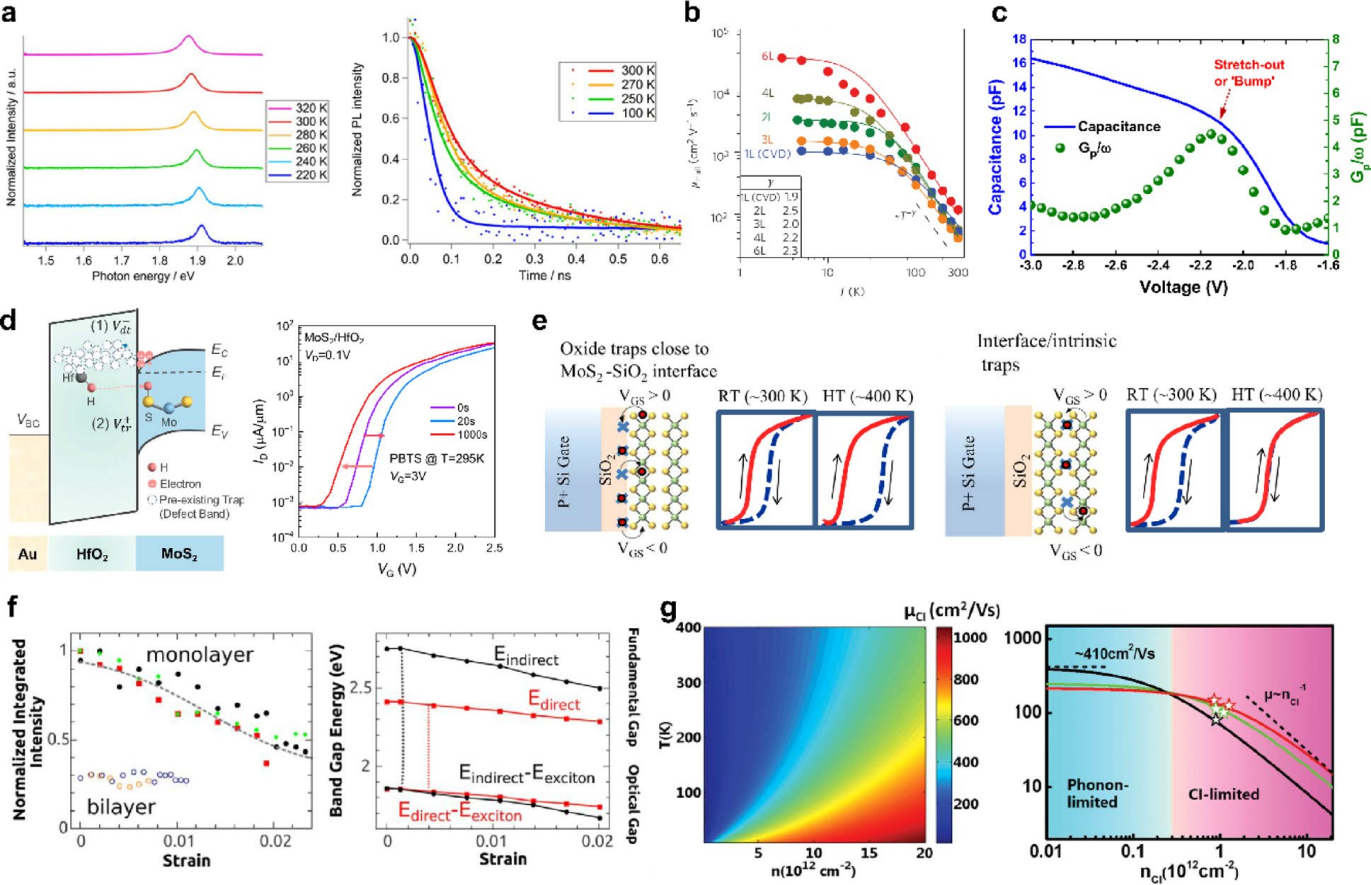
**a** Temperature dependence of PL spectra and a time-resolved PL intensity of hBN/MoS_2_/hBN [164]. **b** Hall mobility of hBN-encapsulated MoS_2_ devices (with different numbers of layers of MoS_2_) as a function of temperature [165]. **c** The depletion capacitance and conductance-voltage (G_p_/ω-V) characteristics measured at a low frequency of 1 kHz in HfO_2_/MoS_2_ capacitor. The stretch-out or bump seen in the C-V curve as a result of interface traps is evidenced by the G_p_/ω peak which unambiguously marks the activity of midgap traps that represents the losses due to the exchange of carriers with the interface traps [169]. **d** Band diagram of 1L-MoS_2_ FETs on HfO_2_ dielectric during positive bias temperature stress (PBTS) and evolution of I_DS_–V_GS_ characteristics under gate bias stress of 1L-MoS_2_ FET under PBTS condition (V_G_ = 3 V) from 0 to 1000 s [40]. **e** Thermally activated oxide traps (close to MoS_2_) that can capture and release electrons from MoS_2_. A negative gate bias releases electrons trapped at the SiO_2_/MoS_2_ interface into the MoS_2_ channel, leading to positively charged traps, whereas a large positively biased gate results in electron capture, resulting in electrically neutral traps. Intrinsic/interface defects/traps in MoS_2_ cause hysteresis primarily at room temperature and collapses at high temperature [176]. **f** Evolution of intensity of the A peak of strained monolayer MoS_2_ (solid shapes) with a calculated intensity (dashed curve) consistent with a degenerate direct and indirect optical band gap at 1.3 ± 0.6% strain. GW_0_ calculations of the fundamental band gaps of strained monolayer MoS_2_, with an expected degeneracy at ∼5% strain. Optical band gap calculated by including the exciton binding energy yields a degeneracy at ∼0.1% [178]. **g** *µ*_CI_ as a function of carrier density and temperature on HfO_2_ substrate (Coulombic impurity density (n_CI_) = 1.0 × 10^12^/cm^2^). Predicted field-effect mobility as a function of n_CI_ for devices on SiO_2_ (black line), Al_2_O_3_ (green line), and HfO_2_ (red line) substrate [182]

When MoS_2_ or WSe_2_ is fully encapsulated by hBN and separated from 3D oxides, the interface is primarily governed by vdW forces. hBN-encapsulated monolayer MoS_2_ devices show room-temperature mobilities almost an order of magnitude higher than SiO_2_-supported devices and much narrower PL and Raman peaks, indicating suppression of charged impurity and surface optical phonon scattering from the oxide (Fig. 11a) [164]. Beyond simple back-gated configurations, multi-terminal hBN-encapsulated MoS_2_ heterostructure devices with graphene contacts exhibit Hall mobilities approaching the intrinsic phonon limit and clear quantum oscillations, directly confirming that the removal of amorphous 3D oxides and rough SiO_2_ substrates strongly suppresses extrinsic charged-impurity and remote-phonon scattering (Fig. 11b) [165]. A similar vdW strategy has recently been extended to 2D perovskite oxide dielectrics, such as Sr_2_Nb_3_O_10_, which can be assembled onto MoS_2_, WS_2_ or WSe_2_. Sr_2_Nb_3_O_10_ combines a high dielectric constant (κ ≈ 25). It can act simultaneously as a photoactive high-κ gate dielectric and vdW insulator, giving MoS_2_ FETs with an on/off ratio of 10^6^, a SS of ~ 80–90 mV/dec, and low leakage at only a few volts of gate bias [47].

For top-gated MoS_2_ and WSe_2_ FETs with Al_2_O_3_ or HfO_2_, the primary interaction is vdW, but nucleation occurs preferentially at defects, edges, or introduced functional groups. McDonnell et al. showed that HfO_2_ deposited on MoS_2_ by ALD forms a conformal high-κ film once nucleation is initiated, as revealed by cross-sectional TEM, AFM, and XPS, with a sharp interface and a negligible interfacial silicate or oxide reaction layer, but with a non-zero density of oxygen-vacancy-related traps in HfO_2_ [166]. In sub-10 nm Al_2_O_3_ gate oxides on MoS_2_ prepared by remote O_2_ plasma pre-treatment, Yang et al. showed that surface activation enables pinhole-free oxides with low leakage and mobility enhancement, but introduces additional interface defect states and strain [52].

In double-gated WSe_2_ FETs with an Al_2_O_3_ or HfO_2_ top-gate stack, the combination of an evaporated Al_2_O_3_ seed and ALD HfO_2_ yields low hysteresis (≈70 mV), demonstrating that carefully engineered bilayer stacks can produce high-κ, electrically clean interfaces even on WSe_2_ [167]. Flexible WSe_2_ FETs with ALD-Al_2_O_3_ top dielectrics on plastic substrates further confirm that vdW semiconductors can withstand repeated bending without interface degradation, provided the Al_2_O_3_/WSe_2_ interface is free of large-area cracks or reaction layers [168].

Electrostatically, a generic vertical gate stack with a 2D channel and oxide can be described by

$$ \frac{d}{dz}\left( {\varepsilon \left( z \right)\frac{d\varphi }{{dz}}} \right) = - \rho \left( z \right), $$

with an interface boundary condition

$$ \varepsilon_{ox} E_{ox} - \varepsilon_{2D} E_{2D} = \sigma_{int} $$

where $\varepsilon \left( z \right)$ is the permittivity, $\varphi$ is the electrostatic potential, $\rho \left( z \right)$ is the volume charge density, $E_{ox}$ and $E_{2D}$ are the electric fields on the oxide and 2D layer sides of the interface, respectively, $\varepsilon_{ox}$ and $\varepsilon_{2D}$ are the permittivities, and $\sigma_{int}$ includes fixed oxide charge, interfacial dipoles and trapped charge. In 2D/3D stacks, $\sigma_{int}$ can reach 10^12^–10^13^/cm^2^, shifting $V_{th}$ by several volts and effectively remote-doping the 2D channel (Fig. 11c) [169, 170].

#### Interface defects and reliability

The basal plane of MoS_2_, WSe_2_, and other TMDs is free of dangling bonds, so that the dominant interface traps at dielectric/TMD interfaces arise from intrinsic defects (e.g., chalcogen vacancies, grain boundaries) with oxide defects (e.g., oxygen vacancies and lattice distortion) in high-κ films near the interface [171]. In practice, ${D}_{it}$ includes both interface states located at the TMDs and border traps in the 3D dielectrics that remain electrostatically coupled to the atomically thin channel (Fig. 11d) [3, 40]. Comparative analyses between scaled Si/SiO_2_ MOSFETs and TMD/high-κ stacks indicate that even optimized MoS_2_ and WSe_2_ FETs still exhibit interface trap densities that are typically one to two orders of magnitude higher than in state-of-the-art Si devices, highlighting the importance of further interface and defect engineering to bring 2D transistor performance closer to Si technology [172].

For a thin-body 2D FET, the impact of these traps on the SS can be written as

$$ SS = \frac{kT}{q}\ln 10\left( {1 + \frac{{C_{it} }}{{C_{ox} }}} \right), $$

$$ C_{it} = qD_{it} , $$

so that

$$ D_{it} = \frac{{C_{ox} }}{q}\left( {\frac{S}{{\left( \frac{kT}{q} \right)\ln 10}} - 1} \right), $$

where $SS$ is the subthreshold swing, $k$ is the Boltzmann constant, $T$ is the absolute temperature, $C_{ox}$​ is the oxide capacitance, $C_{it}$​ is the interface-trap capacitance, and $D_{it}$ is the interface-trap density. Even a modest degradation from the ideal 60 mV/dec to ~ 120 mV/dec corresponds to $D_{it}$ ~ 10^12^/cm^2^·eV for Al_2_O_3_ and HfO_2_ gate stacks on MoS_2_ or WSe_2_, consistent with values extracted from 2D devices [85, 169, 173–175].

Hysteresis and bias-temperature instability in MoS_2_ FETs are well described by an effective trapped charge $Q_{trap} = qN_{trap}$ that induces a threshold-voltage shift $\Delta V_{th}$

$$ \Delta V_{th} \cong \frac{{Q_{trap} }}{{C_{ox} }} = \frac{{qN_{trap} }}{{C_{ox} }}. $$

Temperature-dependent hysteresis and gate-sweep-rate studies on MoS_2_ transistors show that this $\Delta V_{th}$ originates from at least two types of traps with time: interface traps that mainly affect the SS and border/oxide traps that dominate wide hysteresis (Fig. 11e) [176]. Recent reliability work on monolayer MoS_2_ on ultrathin HfO_2_ reveals a two-stage $\Delta V_{th}$ under positive bias temperature stress, with an initial shift attributed to pre-existing oxide traps and a slower component associated with stress-generated donor states in the dielectric, where the effects are strongly suppressed when the same channel is placed on hBN instead of HfO_2_ [40]. Consistently, single-defect spectroscopy in ultrascaled MoS_2_ FETs shows that just a few deep traps with capture/emission times in the millisecond–second range can dominate hysteresis and long-term drift, providing a microscopic origin for the large effective ${D}_{it}$ [177].

Strains also play essential roles. ALD-deposited high-κ layers often induce biaxial strain in TMDs due to thermal-mismatch-induced densification. The in-plane strain shifts the band edges and can modify Schottky barrier heights and contact resistance. In monolayer MoS_2_, uniaxial tensile strain in the ~ 0.5–1% range reduces the band gap by ∼40–50 meV/% and can drive a direct–indirect band-gap crossover, directly impacting carrier injection across metal/TMD contacts (Fig. 11f) [178]. Strain-engineered MoS_2_ FETs on flexible or process-stressed substrates exhibit nearly twofold increases in room-temperature mobility and on-current, consistent with a strain-induced reduction in the effective Schottky barrier height and specific contact resistance [179]. Moreover, electrically controlled strain transfer via piezoelectric thin films in MoS_2_ FETs demonstrates reversible modulation of drain current, on/off ratio, and mobility through minor (~ 0.1–0.2%) biaxial strain, highlighting strain engineering as a complementary knob to conventional contact and dielectric optimization in scaled 2D transistors [180]. These experimental observations are consistent with recent strain-engineering studies of 2D semiconductors, which identify strain as a co-optimized design parameter alongside gate-stack and contact engineering in 2D electronics [181].

#### Scattering mechanisms in 2D/3D stacks

High-κ dielectrics such as HfO_2_ and ZrO_2_ increase gate capacitance and EOT, but their polar surface optical phonons and interfacial charges introduce additional scattering. The effective low-field mobility $\mu_{eff}$ can be expressed as

$$ \frac{1}{{\mu_{eff} }} = \frac{1}{{\mu_{ph} }} + \frac{1}{{\mu_{RP} }} + \frac{1}{{\mu_{CI} }} + \ldots , $$

where $\mu_{ph}$ is the intrinsic phonon-limited mobility, $\mu_{RP}$ is the remote phonon contribution from gate dielectrics, and $\mu_{CI}$ is the charged-impurity-limited contribution. Yu et al*.* and others showed that by combining high-κ HfO_2_ substrates with high carrier concentrations, scattering from charged impurities can be strongly screened, allowing room-temperature mobilities of 150 cm^2^/V·s in monolayer MoS_2_ and approaching the intrinsic phonon-limited regime (Fig. 11g) [182]. Conversely, mobility studies comparing low-κ and high-κ dielectrics suggest that an effective air gap or vdW gap between MoS_2_ and the oxide can mitigate remote phonon scattering and enhance carrier transport closer to the free-standing limit [183].

### Emerging gate dielectrics for 2D FETs

Emerging dielectrics for 2D electronics are moving beyond conventional SiO_2_, Al_2_O_3_, and HfO_2_ to address the simultaneous requirements of sub-nanometer EOT, mechanical flexibility, and new functionalities such as nonvolatile gating (Table 4). Dielectrics must suppress interface traps, minimize remote phonon and Coulomb scattering, tolerate mechanical strain, and often provide additional functions such as non-volatile polarization doping or optoelectronic response. For 2D channels such as MoS_2_, WSe_2_, WS_2_, and MoSe_2_, this has driven the exploration of four classes of emerging dielectrics: high-κ oxides, ferroelectric oxides and nitrides, 2D dielectrics, and polymer and ion-gel dielectrics. In this section, we focus exclusively on dielectrics demonstrated with 2D channels and emphasize interface physics, rather than bulk dielectric properties.

**Table 4:** Comparative summary of dielectric properties and device performance for 2D semiconductor FET gate stacks

Type (TMDs)	Gate dielectric	Dielectric constant	EOT [nm]	I_on_/I_off_	SS [mV/dec]	Dit [cm^−2^*eV]	Breakdown field [MV/cm]	References
MoS_2_	HfO_2_	15	1.5	10^7^	89	NA	NA	[40]
MoS_2_	Al_2_O_3_	8.5	1.38	10^7^	68	7.6 × 10^9^	NA	[19]
MoS_2_	Gd_2_O_5_	25.5	1	10^8^	75	2.2 × 10^12^	6.9–15.7	[184]
MoS_2_	AlScN	NA	NA	10^6^	100	NA	NA	[185]
MoS_2_	Bi_2_SeO_5_	16.5	6.6	10^8^	70	NA	10–30	[186]
MoS_2_	Bi_2_SiO_5_	32	2.7	10^8^	62	NA	9.4	[187]
MoS_2_	ZrO_2_	18.1	0.86	10^8^	75	NA	NA	[188]
MoS_2_	HZO/Al_2_O_3_	NA	4.4	10^7^	57.6	NA	NA	[189]
Bi_2_O_2_Se	Bi_2_SeO_5_	21	0.9	10^5^	75	NA	NA	[190]
HfSe_2_	HfO_2_	23	0.5	10^8^	61	5 × 10^10^	NA	[191]

#### High-κ dielectrics

For scaled 2D TMD-based FETs, high-κ oxides are essential to achieve sub-nm EOT while maintaining low leakage and low interface trap density. Recent work has moved from conventional amorphous HfO_2_ and Al_2_O_3_ toward single-crystalline or quasi-vdW high-κ oxides that better match the layered nature of 2D semiconductors.

**Fig. 12:**
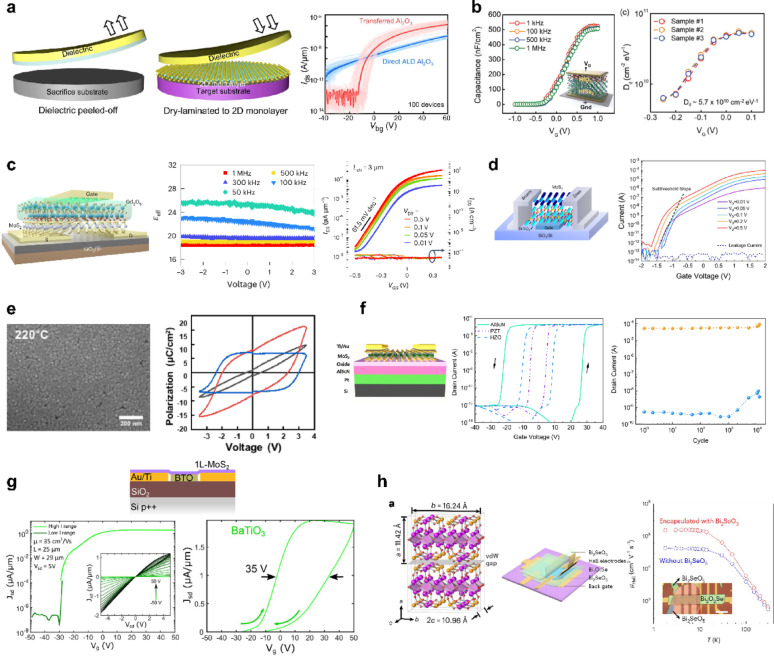
**a** The I_DS_–V_GS_ transfer characteristic of 100 back-gate MoS_2_ transistors with both transferred Al_2_O_3_ and direct ALD Al_2_O_3_ [19]. **b** MOS capacitor based on HfO_2_/HfSe_2_ and quantitative analysis of HfO_2_/HfSe_2_ interface quality: capacitance–voltage characteristics (inset: a structure of HfO_2_/HfSe_2_-based MOS capacitor fabricated with 10 nm HfO_2_ and 15 nm HfSe_2_) and interface charge trap density (D_it_ ≈ 5.7 × 10^10^/cm^2^ eV at 1 kHz) by the conductance method from the measured conductance [191]. **c** Dielectric properties of 2D Gd_2_O_5_. The effective dielectric constant (ε_eff_) of ~ 32 nm Gd_2_O_5_ at various frequencies measured using MIM structure and dual-sweep I_DS_–V_GS_ curves of a MoS_2_ FET using the graphite top electrode under various *V*_DS_ [184]. **d** Schematic of the MoS_2_/STO high-κ 2D FET with bottom-gate configuration, and its normalized I_DS_–V_GS_ characteristics were measured by applying different V_DS_ [192]. **e** Plan-view SEM analysis results for 10-nm-thick as-deposited HZO films on MoS_2_ and its polarization properties of MFS (Mo/HZO/MoS_2_) capacitors measured in the positive-up-negative-down (PUND) mode [193]. **f** Schematic view of an AlScN/MoS_2_ FE-FET. Semilogarithmic scale transfer characteristics at room temperature of a representative AlScN/MoS_2_ FE-FET with 100 nm AlScN as the ferroelectric gate dielectric, comparison with ferroelectric dielectrics of 100 nm thick PZT, HfO_2_, and AlScN [185]. **g** Source-drain current of MoS_2_ FET as a function of the gate voltage for a device integrating a BTO (48 nm)/SiO_2_ (285 nm) dielectric [196]. **h** Crystal structure of Bi_2_SeO_5_ and temperature dependent Hall mobility (μ_Hall_) of 2D Bi_2_O_2_Se with and without Bi_2_SeO_5_ encapsulation [186]

The first group comprises amorphous and polycrystalline high-κ oxides, which are incorporated using state-of-the-art processing techniques. Wafer-scale Al_2_O_3_ and HfO_2_ layers have been dry-transferred onto monolayer MoS_2_ and other 2D channels using vdW integration. This approach enables sub-3 nm physical thickness and EOT ≲ 1 nm, while preserving uniform coverage and low hysteresis in large-area circuits (Fig. 12a) [19]. More recently, a native HfO_2_ gate dielectric has been realized by plasma oxidizing 2D HfSe_2_. The top HfSe_2_ layer is chemically converted into HfO_2_ (κ ≈ 23), forming an atomically sharp HfO_2_/HfSe_2_ interface with ${D}_{it}$ ~ 5 × 10^10^/cm^2^·eV and EOT ≈ 0.5 nm, while keeping gate leakage around 10^−3^ A/cm^2^ (Fig. 12b) [191]. This system demonstrates that high-quality high-κ/2D stacks can be achieved by controlled oxidation of a layered semiconductor itself, rather than depositing other oxides.

Native high-κ oxides formed from 2D semiconductors extend this concept further. Oxidation of Bi_2_O_2_Se produces a single-crystalline β-Bi_2_SeO_5_ layer (κ ≈ 21) epitaxially matched to the underlying 2D semiconductor. Devices using β-Bi_2_SeO_5_ as the gate dielectric exhibit EOTs of ~ 0.4–0.9 nm, leakage currents below the low-power limit, and SS near the thermionic limit, while maintaining high mobility and minimal hysteresis [190]. Here, β-Bi_2_SeO_5_ is a chemically converted native oxide that serves simultaneously as a high-κ dielectric and a structurally coherent cap for Bi_2_O_2_Se and potentially other 2D channels.

High-κ single-crystalline 3D oxides are also being adapted to 2D electronics. Gd_2_O_5_ grown as a 2D single-crystalline dielectric by vdW epitaxy combines a large κ with a wide bandgap, enabling sub-1 nm EOT and low-power 2D nanoelectronics when integrated with vdW semiconductors (Fig. 12c) [184]. Although Gd_2_O_5_ is not itself a layered material, its epitaxial growth on a quasi-vdW substrate yields interfaces with 2D channels that approach the ideality of fully layered stacks.

As a perovskite oxide, SrTiO_3_ (STO), a cubic perovskite with ultrahigh κ (≈ 200–300 at room temperature), has recently been employed as a single-crystalline vdW gate dielectric for MoS_2_ FETs. STO/MoS_2_ devices exhibit small hysteresis, a low SS (~ 70–80 mV/dec), and improved stability under bias stress, demonstrating that an epitaxial perovskite oxide can serve as an ultrahigh-κ gate while still forming a relatively poor interface with a 2D semiconductor [192].

#### Ferroelectrics

Ferroelectric dielectrics coupled to 2D semiconductors enable nonvolatile charge modulation and multilevel memory states through their remanent polarization $P_{FE}$. The polarization surface charge density $\sigma_{FE} = \pm P_{FE}$ changes the channel carrier density $\Delta n$ by.

$$\Delta n = \frac{{P_{FE} }}{q}$$,

and produces a threshold-voltage shift.

$$\Delta V_{th} = \frac{{P_{FE} }}{{C_{ox} }}$$,

where $C_{ox}$ is the capacitance of the ferroelectric material. In MoS_2_ and WSe_2_ ferroelectric FETs (FeFETs) using HZO or related hafnia-based ferroelectrics, memory windows of 1–2 V and on/off ratios of 10^5^–10^6^ have been reported, which are compatible with scaled gate lengths. Direct growth of HZO on MoS_2_ shows that careful control of the interface can suppress interfacial reactions and maintain robust ferroelectric switching, making HZO a promising BEOL-compatible ferroelectric for 2D logic and memory (Fig. 12e) [193].

Wurtzite Scandium-doped AlN (AlScN) has recently emerged as a CMOS-compatible ferroelectric nitride that can be sputter-deposited at relatively low temperatures. AlScN/2D-channel FeFETs with MoS_2_ and WSe_2_ channels demonstrate large memory windows, endurance beyond 10^6^ cycles, and retention exceeding 10^4^ s, while operating at sub-5 V gate voltages (Fig. 12f) [185]. High-current AlScN/MoS_2_ FeFETs further demonstrate that polarization charge can support carrier densities above 1014/cm^2^ and on-currents exceeding 1 mA/µm, suggesting that ferroelectric gating can compensate for the relatively low density of states in ultrathin 2D channels [194].

Perovskite ferroelectrics such as BaTiO_3_ and related titanates have been incorporated into MoS_2_ channels to realize optoelectronic FeFETs and memristive devices, in which ferroelectric domains modulate both the Schottky barrier and local band bending in the TMD [195]. Freestanding BaTiO_3_ layers can be integrated with MoS_2_ to form ferroelectric gate stacks in which the MoS_2_ channel conductivity, memory window, and Schottky barrier height are reversibly tuned by polarization (Fig. 12g) [196]. These studies indicate that ferroelectric oxides and nitrides can be co-integrated with MoS_2_, WSe_2_, and related 2D semiconductors to realize nonvolatile gating, steep transfer characteristics, and high-density charge modulation without fundamentally changing the channel material. Beyond purely electrical control, recent studies have explored alternative mechanisms such as mechanically induced polarization switching and reconfigurable ferroelectric-gated device functionalities in 2D systems [197, 198].

#### 2D dielectrics

hBN is the conventional 2D dielectric for 2D electronics. Its wide band gap of ~ 6 eV, dielectric constant of 3–4, and atomically flat, dangling-bond-free basal plane provide an electronically clean environment that strongly suppresses charged-impurity and surface-phonon scattering compared with SiO_2_. hBN substrates and encapsulation layers have enabled high-mobility graphene and TMD devices with reduced hysteresis and narrow spectral linewidths, underscoring the importance of all-2D gate stacks for probing intrinsic transport and optics in MoS_2_ and WSe_2_ [164, 199, 200].

However, the relatively low κ of hBN limits electrostatic control when the equivalent oxide thickness is aggressively scaled, motivating the search for new vdW dielectrics with higher permittivity but similarly clean interfaces. Single-crystalline Bi_2_SeO_5_ has recently emerged as a high-κ (κ ≈ 16–20) layered dielectric that can be exfoliated into atomically flat nanosheets and integrated via vdW assembly (Fig. 12h) [186, 201]. When used as both the gate insulator and the encapsulation layer for MoS2 and graphene channels, Bi_2_SiO_5_ substantially enhances mobility and enables accumulation of carrier densities > 10^13^ cm^−2^ at gate biases of only a few volts, demonstrating that vdW high-κ dielectrics can deliver strong electrostatic control without sacrificing interface quality.

A complementary direction is Bi_2_SiO_5_, another layered bismuth oxy-compound that can be synthesized as ultrathin single crystals by CVD and transferred onto arbitrary substrates [187]. Vertically grown Bi_2_SiO_5_ nanoplates exhibit an effective permittivity κ ≈ 30–35 over a broad thickness range, together with a large optical band gap (~ 3.8 eV) and breakdown fields on the order of 7–9 MV/cm. As a dielectric for few-layer MoS_2_ FETs, Bi_2_SiO_5_ affords capacitance over 1 µF/cm^2^, enabling large carrier modulation, near-ideal SS (~ 60–70 mV/dec), and small hysteresis even in short-channel devices. These results show that all-vdW gate stacks based on high-κ 2D dielectrics can combine strong electrostatic control with low trap densities, offering an attractive alternative to conventional HfO_2_ or Al_2_O_3_ while maintaining the structural and chemical compatibility required for scalable 2D MoS_2_ and WSe_2_ electronics.

#### Polymer dielectrics

Polymer dielectrics are not directly coupled to ALD process chemistry. Yet, they are highly relevant in dielectrics on 2D materials because their electrical behavior is also governed by the dielectric/2D interface and by interfacial charge formation at a vdW contact. Polymer dielectrics provide mechanically soft, chemically versatile gate insulators that are particularly attractive for flexible and stretchable 2D electronics. In 2D FETs based on MoS_2_, WSe_2_, and related TMDs, polymers appear mainly in two forms: (i) ion gels or solid polymer electrolytes that form an electric double layer (EDL) at the 2D surface, and (ii) ferroelectric polymers that supply nonvolatile polarization charge. In both cases, the vdW interface between the 2D semiconductor and the polymer is generally chemically benign compared to that of conventional high-κ oxides. Still, the presence of mobile ions or dipoles introduces time-dependent characteristics and reliability considerations.

**Fig. 13:**
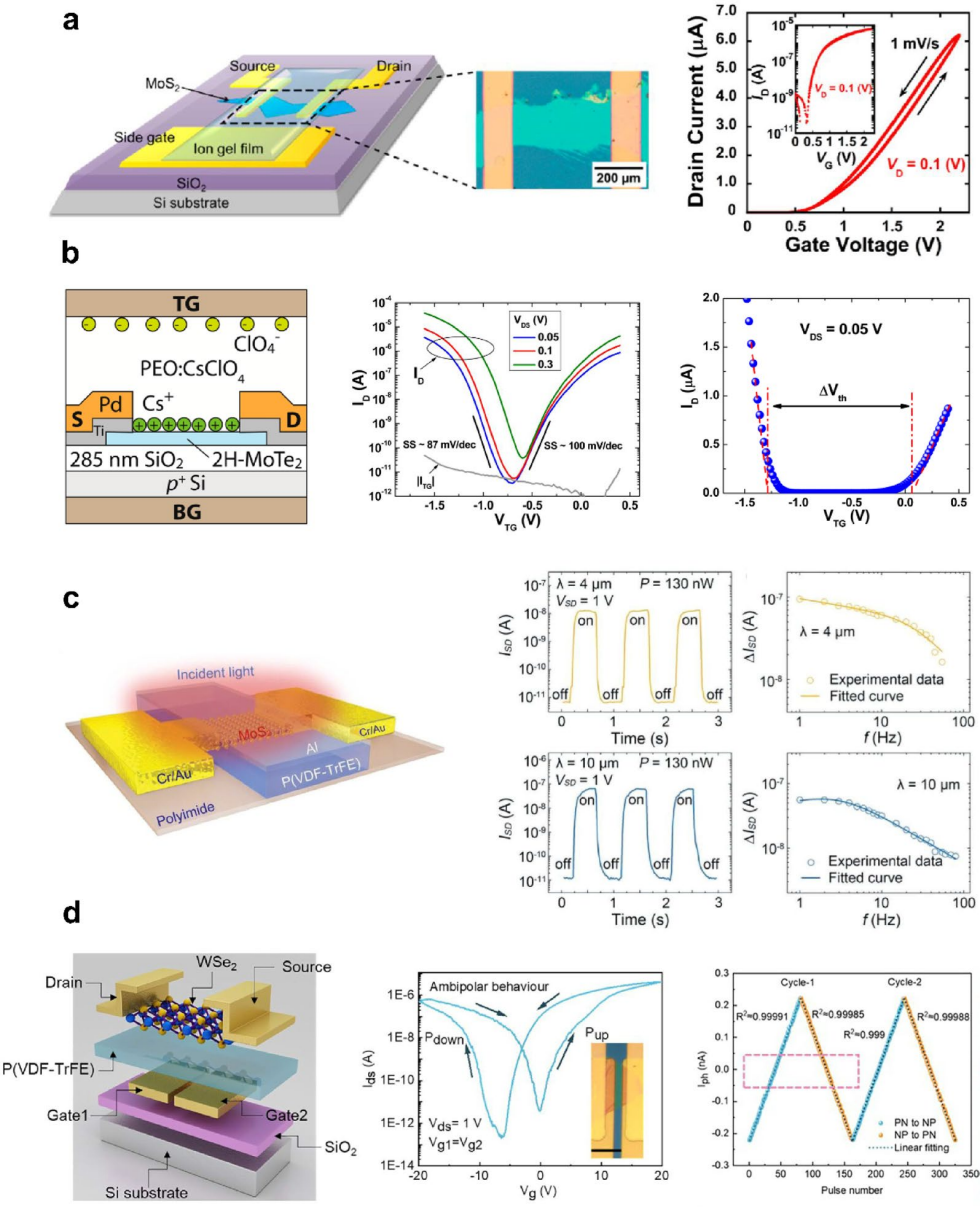
**a** Schematic depiction and optical image of the MoS_2_ electric-double-layer transistor (EDLT) and its transfer characteristics [202]. **b** Room temperature current–voltage characteristics of a PEO:CsClO_4_ solid polymer electrolyte-gated 2H-MoTe_2_ FET [204]. **c** Photocurrent switching behavior of the MoS_2_/PVDF-TrFE device with MIR light at V_DS_ = 1 V and the photocurrent response (ΔI_DS_) dependence of the photodetector as a function of frequency under infrared light [205]. **d** 3D image of the WSe_2_/P(VDF-TrFE) transistor, its transfer curve under equivalent voltages of the two gates, and bidirectional potentiation and depression processes with high linearity and symmetry [207]

Pu et al. demonstrated that MoS_2_ thin-film transistors gated by ion gels operate at < 1 V, with mobilities of ~ 12.5 cm^2^/V·s, on/off ratios of ~ 10^5^, and excellent mechanical flexibility, highlighting the compatibility of soft ionic dielectrics with flexible MoS_2_ channels (Fig. 13a) [202]. Building on this concept, Wu et al. used an electrolyte gate to achieve ambipolar MoS_2_ transport with SS < 50 mV/dec and ultra-high carrier density (~ 10^14^/cm^2^), enabling tunable photoresponse and reconfigurable p–n homojunction behavior in a single MoS_2_ device [203]. Additionally, Xu et al. showed that poly(ethylene oxide)/CsClO_4_ solid polymer electrolytes can electrostatically dope in 2H-MoTe_2_ FETs between n- and p-type operation, with measured EDL capacitances ~ 4 µF/cm^2^ and maximum carrier density ~ 1.6 × 10^13^/cm^2^, illustrating how polymer electrolytes can serve as powerful, reconfigurable gates for vdW semiconductors (Fig. 13b) [204].

Ferroelectric polymers such as P(VDF–TrFE) offer a complementary route to oxide and nitride ferroelectrics, combining large remanent polarization with low-temperature, solution-processable integration on 2D semiconductors. In TMDs/P(VDF–TrFE) hybrid devices, the polarized polymer layer acts simultaneously as a high-κ gate and a nonvolatile charge reservoir: downward (upward) polarization electrostatically accumulates (depletes) electrons in TMDs, shifting the transfer curve and modulating the channel conductance. Wang et al. showed that quasi-freestanding MoS_2_ on P(VDF–TrFE) exploits this effect to suppress dark current, enhance responsivity, and maintain a persistent change in photocurrent after removal of the gate bias, demonstrating that the ferroelectric polarization can serve as a built-in, nonvolatile gate for 2D channels (Fig. 13c) [205]. Recent FeFET roadmaps that explicitly include 2D semiconductors identify P(VDF–TrFE) and related copolymers as key candidates for flexible, back-end-of-line-compatible 2D memory and neuromorphic devices, because their polarization can be reversed with sub-10 V pulses and retained for > 10^4^–10^5^ s without significant drift [206]. A WSe_2_/P(VDF–TrFE) transistor architecture uses locally poled ferroelectric domains to write nonvolatile p–n and n–p homojunctions along a single WSe_2_ channel, yielding programmable, bidirectional photocurrents that function as multi-level synaptic weights (Fig. 13d) [207]. Polarization-defined junctions remain stable at zero gate bias, enabling self-powered image sensing and motion detection with high accuracy while avoiding continuous power dissipation.

### Engineered gate-stack design for 2D FETs

Engineering the gate stack provides an additional degree of freedom beyond the selection of a high-quality dielectric for 2D semiconductors. By combining interface physics and emerging dielectrics, multilayer high-κ/low-κ stacks, inserted interfacial layers, charge-trap, and fully 2D/2D gate stacks can be integrated to decouple electrostatics, interface quality, and nonvolatile functionality.

#### High-κ/low-κ multilayers and interfacial layers

In ultra-scaled 2D FETs, a single high-κ layer rarely meets all constraints for low EOT, low interface-trap density, and mechanical/chemical stability. A common strategy is to use a high-κ dielectric to provide large gate capacitance in series with a thin low-κ or interfacial layer that passivates the TMD surface. In the simplest approximation, the equivalent oxide capacitance is

$$ \frac{1}{{C_{eq} }} = \frac{1}{{C_{int} }} + \frac{1}{{C_{HK} }}, $$

where $C_{eq}$ is the series equivalent capacitance, $C_{int}$​ is the capacitance associated with the interfacial (low-κ) layer, and $C_{HK}$ is the capacitance of the high-κ layer. Inserting an interfacial layer inevitably increases the effective EOT. Still, it can simultaneously and substantially reduce $D_{it}$ and the fixed charge, thereby stabilizing the threshold voltage and suppressing hysteresis.

**Fig. 14:**
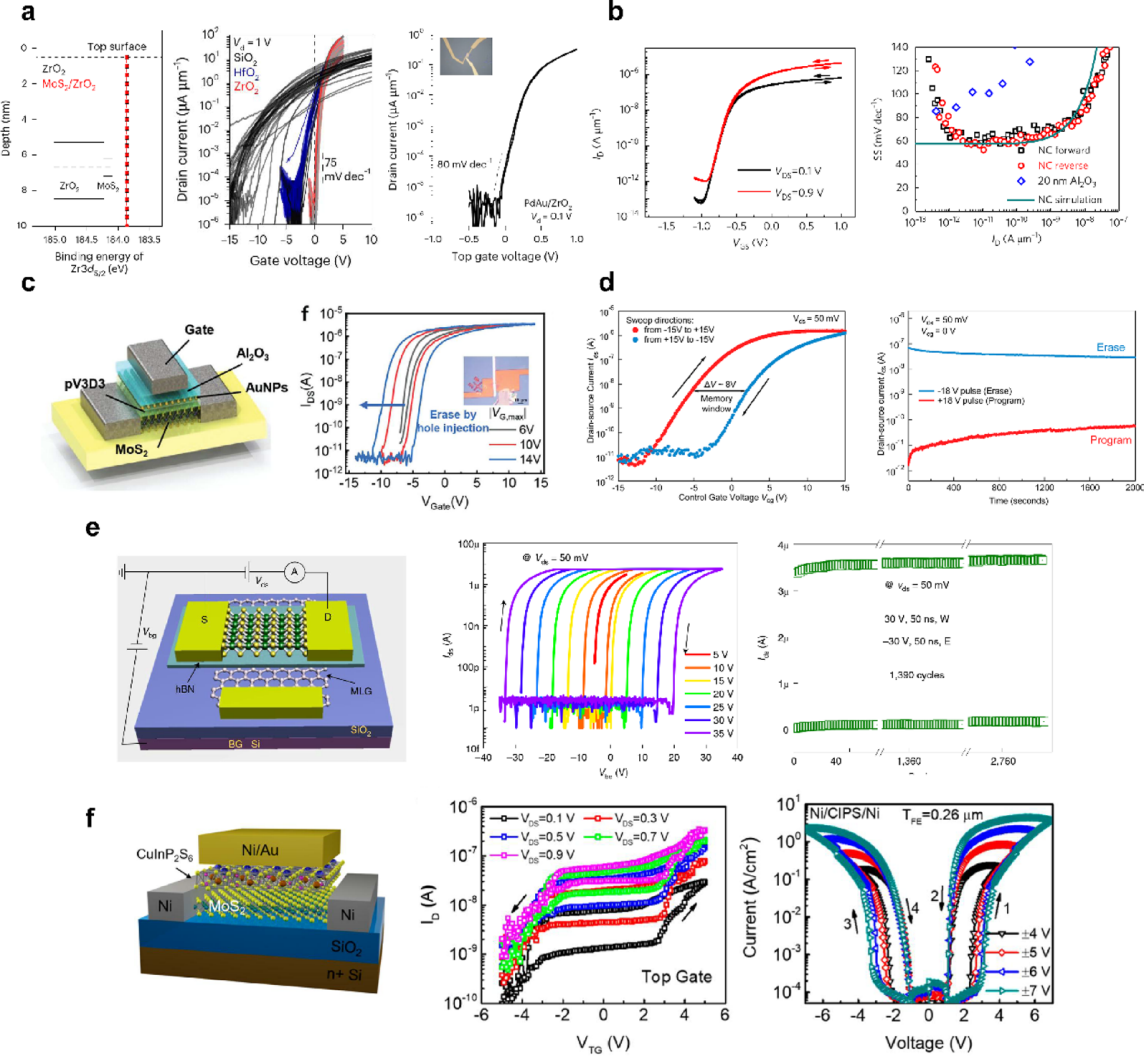
**a** Binding energy positions of the Mo 3d_5/2_ at different depths for ZrO_2_ and MoS_2_ on ZrO_2_, logarithmic transfer characteristics of MoS_2_ FETs showing stable and low SS in devices with a ZrO_2_ dielectric compared with those with SiO_2_ and HfO_2_ dielectrics, and transfer characteristics of top-gated FETs based on a multilayer MoS_2_ and ZrO_2_ as the top-gate dielectric, and PdAu as the top-gate metals [188]. **b** I_DS_–V_GS_ characteristics measured at room temperature and their SS versus I_DS_ characteristics of the MoS_2_/HZO/Al_2_O_3_ device, showing minimum SS below 60 mV/dec for both forward and reverse sweeps [189]. **c** Schematic illustration of the MoS_2_-based synaptic memory device, which is composed of pV3D3 tunneling layer, AuNPs charge storage layer, and Al_2_O_3_ blocking layer, and its electrical characteristics [209]. **d** Schematics of a heterostructure memory cell with a single-layer MoS_2_ semiconducting channel, graphene contacts, and multilayer graphene (MLG) floating gate. The MLG floating gate is separated from the channel by a thin tunneling HfO_2_ and from the control gate by a thicker blocking oxide. Electrical characteristics of the floating gate transistor, acquired along two different control-gate voltage sweep directions [210]. **e** Device structure modulated by the back gate electrode in MoS_2_/hBN/Graphene/SiO_2_ structure and its transfer characteristics and endurance [211]. **f** Schematic view of a MoS_2_/CIPS 2D heterostructure Fe-FET and its electrical characteristics [213]

Recent work on ZrO_2_/MoS_2_ gate stacks illustrates this trade-off in an industry-relevant material system. Yan et al. showed that ALD ZrO_2_ can form an atomically clean, vdW-like interface with monolayer MoS_2_, yielding back-gated and top-gated devices with EOT of 0.86 nm, SS of 75–80 mV/dec, and minimal unintentional doping compared with SiO_2_ or HfO_2_ on the same channel (Fig. 14a) [188]. This result highlights that carefully selected high-κ dielectrics can serve as self-passivating interfacial layers when their chemistry suppresses charge transfer into the 2D channel. Ferroelectric hafnia-based stacks provide a more extreme example of multilayer engineering. In steep-slope MoS_2_ transistors, Si et al*.* employed an Al_2_O_3_/HZO bilayer in the gate stack to match capacitance and suppress leakage through the polycrystalline HZO layer, enabling sub-60 mV/dec switching and nearly hysteresis-free operation (Fig. 14b) [189]. Although motivated by the negative-capacitance (NC) effect, this architecture epitomizes how adding thin interfacial layers can decouple ferroelectric switching, leakage, and interface quality, and the same design concepts are directly transferable to non-NC HZO-based FeFETs on MoS_2_ or WSe_2_.

#### Charge-trap and floating-gate stacks

For nonvolatile memory and neuromorphic applications, the gate stack is deliberately engineered to store charge. A canonical MoS_2_ charge-trap memory consists of a 2D channel, a tunneling dielectric, a charge-trap layer, a blocking dielectric, and a control gate [208]. In such devices, the program/erase window is governed by the gate-coupling ratio

$$ \alpha = \frac{{C_{cg} }}{{C_{cg} + C_{ct} }}, $$

where $C_{cg}$ is the capacitance between the control gate and the 2D channel through the blocking dielectric, and $C_{ct}$ is the capacitance associated with the charge-trap (or floating-gate) layer. A larger $\alpha$ increases the fraction of the applied gate voltage that effectively modulates the channel potential. Therefore, optimizing these capacitances, together with the density and energy distribution of trap states in the charge-trap layer, is essential to achieve large memory windows at low program/erase voltages and high retention.

Kim et al. recently demonstrated a low-power charge-trap flash memory with a MoS_2_ channel tailored for 3D NAND-compatible, in-memory computing (Fig. 14c) [209]. By combining a low-κ tunneling layer with an engineered charge-trap stack, they achieved a large memory window of 8 V, multilevel states, and improved endurance at reduced operating voltages, illustrating how gate-stack design can be co-optimized with 2D channel properties for system-level AI accelerators. Earlier, Bertolazzi et al. integrated a multilayer graphene floating gate above a monolayer MoS_2_ channel, separated by an Al_2_O_3_/HfO_2_ tunneling oxide, thereby realizing an all-2D floating-gate memory cell with a conductance ratio of 10^4^ between the program and erase states (Fig. 14d) [210]. These results emphasize that 2D channels are susceptible to stored charge in nearby layers, enabling large memory windows even when the charge-trap layer is several nanometers away.

#### 2D/2D gate stacks

2D/2D gate stacks, in which the channel, dielectric, and often the gate/floating gate vdW materials, are aimed at approaching an almost ideal interface for 2D electronics. Fully vdW flash-type structures, such as MoS_2_/hBN/multilayer graphene stacks, advanced this idea. In a MoS_2_/hBN/multilayer-graphene flash memory, few-layer hBN acts as a crystalline tunnelling and blocking dielectric between the 2D channel and 2D floating gate, enabling ultrafast 20 ns program/erase times, on/off ratios of 10^6^–10^8^, and projected 10-year retention (Fig. 14e) [211]. Two-terminal MoS_2_/hBN/graphene tunnelling RAM (TRAM)-type cells further simplify the architecture by using the same 2D heterostructure for both gating and current flow, illustrating how vdW assembly can integrate memory and transistor functions into a compact vertical stack [212].

Functional 2D dielectrics add another dimension. MoS_2_ ferroelectric FETs using CuInP_2_S_6_ (CIPS) as a 2D ferroelectric gate insulator create a 2D/2D semiconductor/insulator interface without dangling bonds; these MoS_2_/CIPS FeFETs exhibit evident ferroelectric hysteresis, nonvolatile threshold-voltage shifts, and stable room-temperature operation, confirming that ferroic order can be incorporated directly into an all-vdW gate stack (Fig. 14f) [213].

Despite advantages such as low interface-trap density, suppressed remote-phonon scattering, and excellent mechanical robustness, the approach has clear limitations. Established 2D dielectrics, such as hBN, exhibit only moderate κ, which constrains ultimate EOT scaling, and large-area growth of high-quality 2D dielectrics and 2D ferroelectrics compatible with CMOS back-end processing remains immature. In practice, near-term scalable MoS_2_ and WSe_2_ will likely rely on hybrid stacks that place a 2D dielectric or 2D ferroelectric directly on the channel to preserve interface quality, while using high-κ oxides farther away from the channel to recover electrostatic scaling.

## Device-level perspective: performance, reliability, and applications

From the perspective of 2D-material-based transistors, the gate dielectric plays a crucial role in device performance, reliability, and application potential, extending well beyond that of a simple insulating layer. Owing to their atomic-layer-scale channel thickness, gate electrostatics, which are closely related to gate dielectrics, exert a particularly strong influence on carrier transport and switching behavior. As a result, the quality of the dielectric/2D interface and the gate structure have a much greater impact on device characteristics than in conventional bulk semiconductor devices [10].

### Impact on transistor performance

The switching performance of 2D material-based transistors is strongly governed by the gate dielectric with their impact depending on gate architecture. Different gate configurations include bottom-gate, top-gate, and dual-gate structures, each offering distinct trade-offs in electrostatic control, interface quality, and process complexity. Bottom-gate devices typically benefit from process simplicity and relatively stable interfaces, but often provide less flexible channel modulation in scaled devices due to geometrically constrained gate overlap near the source and drain regions. This limitation can constrain scalability and complicate optimization in densely integrated circuits [214, 215].

**Fig. 15:**
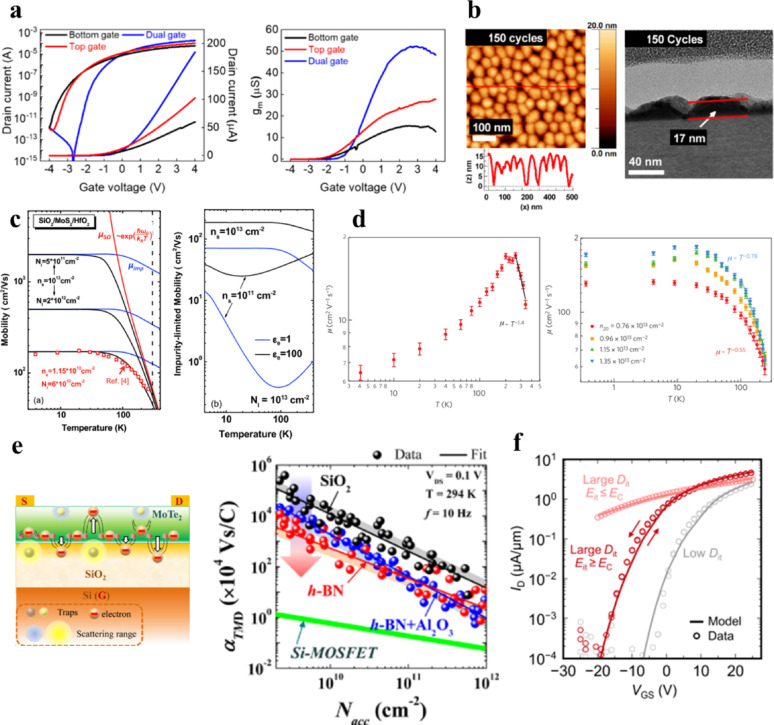
**a** Transfer characteristics and corresponding transconductance of MoS_2_-channel graphene-electrode TFTs measured under bottom-gate(black), top-gate(red), and dual-gate(blue) operation [216]. **b** AFM and cross-sectional TEM images of the HfO_2_/MoS_2_ stack after 150 ALD cycles, revealing non-uniform dielectric nucleation and thickness variation due to the chemically inert surface of MoS_2_ [166]. **c** Temperature-dependent electron mobility in the SiO_2_/MoS_2_/HfO_2_ structure, illustrating mobility degradation induced by remote surface optical phonon interactions and Coulomb scattering [217]. **d** Electron mobility limited by interfacial Coulomb scattering and remote phonon interactions, which give rise to non-ideal temperature and field dependence of transport [48]. **e** Schematic illustration and the interfacial Coulomb-scattering parameter in MoTe_2_ TFTs compared to the conventional bulk semiconductor devices [219]. **f** Transfer characteristics of a MoS_2_ TFT before (gray) and after (light red) AlO_x_ deposition, illustrating the impact of dielectric-induced interface states on device performance [70]

In contrast, the top-gate architecture can provide more direct electrostatic coupling between the gate dielectrics and the channel, enabling finer electrostatic control of the channel potential [215]. This enhanced control minimizes parasitic components, which is important for maintaining device performance at scaled dimensions. Further improvement in electrostatic control can be achieved by extending the gate architecture to dual-gate configurations, where simultaneous modulation from both sides of the channel provides even stronger gate control and enhanced transconductance (Fig. 15a) [216]. However, the direct deposition of gate dielectrics on 2D materials introduces substantial challenges. Many 2D surfaces are chemically inert, leading to poor nucleation during dielectric deposition and the formation of interface traps or physical damage to the channel (Fig. 15b) [19, 166]. Through remote phonon interactions and Coulomb scattering, these interface-related problems can drastically reduce carrier mobility (Fig. 15c) [217]. To mitigate these issues and achieve the desired characteristics, top-gate transistors often require more complex and carefully controlled fabrication processes.

As a consequence of dielectric–channel interactions, carrier mobility, which affects 2D transistor performance, is highly sensitive to dielectric-induced scattering mechanisms. Although high-κ dielectrics are useful for decreasing EOT, they also cause remote phonon scattering, which can restrict mobility, especially at high carrier densities [48]. As a result, room-temperature mobilities in top-gated monolayer devices are often limited to below ~ 100 cm^2^/V·s [2, 184]. Coulomb scattering is further influenced by fixed charges, interfacial dipoles, and trapped charges in the dielectric, resulting in non-ideal temperature- and field-dependent transport properties (Fig. 15d) [48, 217]. Thus, while high-κ dielectrics are attractive for enhancing gate capacitance and channel controllability, they can simultaneously introduce dielectric-induced scattering mechanisms that degrade carrier mobility in atomically thin channels.

Beyond carrier mobility, the gate dielectric and its interface with the 2D channel also play critical roles in determining key switching metrics, such as SS and on/off current ratio. In principle, a steeper SS is enabled by EOT scaling and high gate capacitance with high-κ dielectrics, which improve subthreshold behavior by shifting the channel potential. However, even in devices with scaled gate dielectrics, the achievable SS remains limited by trap-assisted charge exchange at the dielectric/2D interface [218]. Interface trap densities on the order of 10^12^ cm^−2^·eV^−1^ can readily broaden the subthreshold region, resulting in swings of ~ 80–100 mV/dec in atomically thin channels [10]. In atomically thin channels, where the channel potential is extremely sensitive to localized charge fluctuations, these effects are especially noticeable (Fig. 15e) [219]. Similarly, the on/off current ratio is determined not only by the intrinsic band structure of the 2D material but also by dielectric-induced leakage pathways. Dielectric-induced scattering reduces the on-state current, while trap-assisted tunneling–induced gate leakage increases the off-state current, collectively degrading the achievable on/off current ratio (Fig. 15f) [70, 81, 220].

### Scaling, leakage, and variability

In 2D material-based transistors, dielectric scaling is primarily pursued to reduce the EOT and thereby enhance gate-channel coupling [221]. The applied gate voltage in a MOS structure is distributed across a series combination of the oxide capacitance, the channel charge response, the interface-trap capacitance, and the quantum capacitance, which becomes particularly relevant in scaled 2D systems [222]. In conventional bulk devices, a depletion region forms beneath the channel, and the resulting volumetric space charge partially screens the gate electric field. This screening leads to a relatively smooth potential distribution. Atomically thin 2D channels behave very differently. Because a conventional depletion region cannot form, charge responds predominantly through sheet charge modulation [223]. As a result, the channel potential is directly coupled to the dielectric properties and interfacial states. While this behavior allows EOT scaling to have a stronger impact than in bulk devices, it also makes 2D transistors far more sensitive to dielectric defects and interfacial charges [10].

To reduce EOT in 2D transistors, several experimental strategies have been proposed. The most widely adopted approach involves depositing high-κ dielectrics by ALD, often combined with surface functionalization or the introduction of seed layers to overcome the lack of dangling bonds on pristine 2D surfaces. Using such methods, sub-nanometer EOT values have been achieved, leading to improvements in SS and enhanced reliability in scaled devices [98]. Beyond amorphous ALD dielectrics, crystalline or semicrystalline ultrathin oxide dielectrics with low defect densities have emerged as promising alternatives that simultaneously achieve EOT scaling and improved interface quality [41, 224]. In addition, vdW-based stacking approaches, in which pre-formed dielectric thin films are transferred onto 2D channels, have been proposed to minimize process-induced damage from plasma or chemical precursors while ensuring wafer-scale thickness uniformity [19, 225, 226].

Despite these advances, aggressive EOT scaling inevitably increases gate leakage current. As the dielectric thickness approaches the nanometer regime, direct tunneling becomes the dominant transport mechanism, and leakage current rises exponentially [227]. Real dielectrics introduce further complexity. Defect-assisted processes such as trap-assisted tunneling, Poole–Frenkel emission, and Fowler–Nordheim tunneling under high electric fields all contribute to leakage conduction [228]. In 2D devices, this issue is particularly severe. Because the channel resides directly beneath the dielectric, localized electric-field enhancement near defects can directly influence the channel potential. Off-state leakage increases, and subthreshold characteristics degrade. Recent studies have shown that gate leakage can be suppressed while preserving strong gate coupling by employing vdW-engineered dielectrics and 2D interfaces, or low-defect-density high-κ dielectrics [226]. These results highlight an important point, which is that leakage behavior is governed not only by dielectric thickness but also critically by defect density and interface quality.

Statistical variability represents another fundamental challenge associated with dielectric scaling in 2D transistors. Owing to the absence of volumetric charge averaging, even a small number of trapped charges within the dielectric or at the interface can induce threshold voltage shifts [229]. As EOT is further reduced, device-to-device variability becomes increasingly dominated by the stochastic distribution of defects and thickness nonuniformity in ultrathin dielectrics. To mitigate these effects, interface-engineered dielectric stacks and low-defect-density dielectric integration strategies have been widely explored. Such approaches aim to reduce charge trapping and suppress hysteresis, thereby improving threshold voltage stability and enhancing uniformity across large-area device arrays [40].

Overall, EOT scaling in 2D transistors improves gate coupling and short-channel behavior, but simultaneously amplifies the impact of dielectric defects and interfacial charge dynamics. This trade-off motivates a systematic discussion of the reliability and long-term stability of dielectric and 2D stacks.

### Reliability and stability of dielectric/2D stacks

Dielectric reliability is governed by the time-dependent generation and accumulation of defects under continuous electric-field stress, ultimately leading to the formation of conductive paths or dielectric breakdown under critical conditions. Time-dependent dielectric breakdown (TDDB) is commonly described as a multistage process. It begins with an initial transient current response associated with charge trapping and detrapping at pre-existing defects, followed by a gradual increase in leakage current driven by stress-induced defect generation [230]. Breakdown occurs when these defects form a percolative conduction path across the dielectric. During this evolution, trap-assisted tunneling (TAT) often emerges as the dominant leakage mechanism. Stress-generated defects cause it and is frequently observed as a precursor to soft breakdown, preceding the formation of a fully conductive channel. When the defect path develops into a sufficiently low-resistance conduction channel, local Joule heating accelerates current flow. This transition leads to a hard breakdown, typically accompanied by thermal runaway [231].

Because 2D channels are atomically thin and in direct contact with the gate dielectric, defects generated within the dielectric or near the interface are directly reflected in channel modulation and leakage characteristics [17]. In the absence of volumetric charge averaging, localized electric field enhancement near defects further accelerates TDDB by increasing TAT conduction. This effect is particularly pronounced in ultrathin high-κ dielectrics, where the critical conditions for defect percolation are relaxed. As a result, leakage current increases, and performance degradation can be observed even before a clearly defined breakdown event occurs. TDDB lifetime and its statistical characteristics can be improved through vdW-based dielectric integration or by using insulating materials with relatively low defect densities.

Bias temperature instability (BTI), hysteresis, and long-term threshold voltage drift arise from charge trapping and detrapping at defects at the dielectric/2D interface and in the dielectric bulk. Under combined gate bias and thermal stress, the occupation state of these traps evolves, leading to threshold voltage shifts and irreversible changes in transfer characteristics [40]. In 2D transistors, the effective gate capacitance, which is connected in series with the quantum capacitance, is reduced. Consequently, a given amount of trapped charge induces a disproportionately large shift in threshold voltage, making BTI and hysteresis effects more pronounced in 2D transistors. This behavior is particularly evident in thin-film transistor configurations. Large-area channels and low-temperature-processed dielectrics often exhibit broad trap energy distributions, leading to cumulative effects under prolonged bias stress that dominate device behavior. As a result, hysteresis becomes highly dependent on measurement conditions, and long-term threshold voltage drift limits device reproducibility and circuit stability. Interface engineering and vdW-based dielectric stacks have therefore been explored as effective approaches to suppress charge trapping and mitigate BTI and long-term threshold voltage drift [110].

Environmental stability represents another critical factor influencing the reliability of 2D transistors. Moisture and oxygen readily adsorb onto exposed channel surfaces, inducing charge-transfer doping and forming interfacial traps. These processes lead to threshold voltage instability and increased hysteresis. Optical illumination can further exacerbate instability through photogating effects and accelerated trap detrapping, while thermal stress modifies adsorption–desorption equilibria and trap emission time constants [232]. When combined with electrical stress, such environmental factors tend to accelerate both TDDB and BTI degradation mechanisms. To mitigate environmentally induced degradation, various strategies have been explored. These include channel passivation, the use of chemically stable dielectrics, and the reduction of residual species introduced during processing. Functional polymer passivation layers and vdW-based stacking architectures have been reported to reduce sensitivity to ambient exposure effectively and to maintain stable electrical characteristics under long-term operation [233].

In summary, dielectric scaling in 2D semiconductor transistors inherently involves a delicate balance among enhanced gate coupling, increased leakage susceptibility, and amplified variability. While EOT reduction is essential for suppressing short-channel effects, scalable and reliable device operation requires simultaneous control of not only dielectric thickness but also defect density and interfacial charge dynamics.

### Application-oriented device examples

In practical 2D material–based devices, the role of the gate dielectric is rarely universal and is often dictated by the intended application. While dielectric scaling and interface quality are critical to performance and reliability, many device concepts tolerate, or even rely on, dielectric-related non-idealities. As a result, gate stack requirements diverge significantly across logic, memory, and emerging functional devices.

**Fig. 16:**
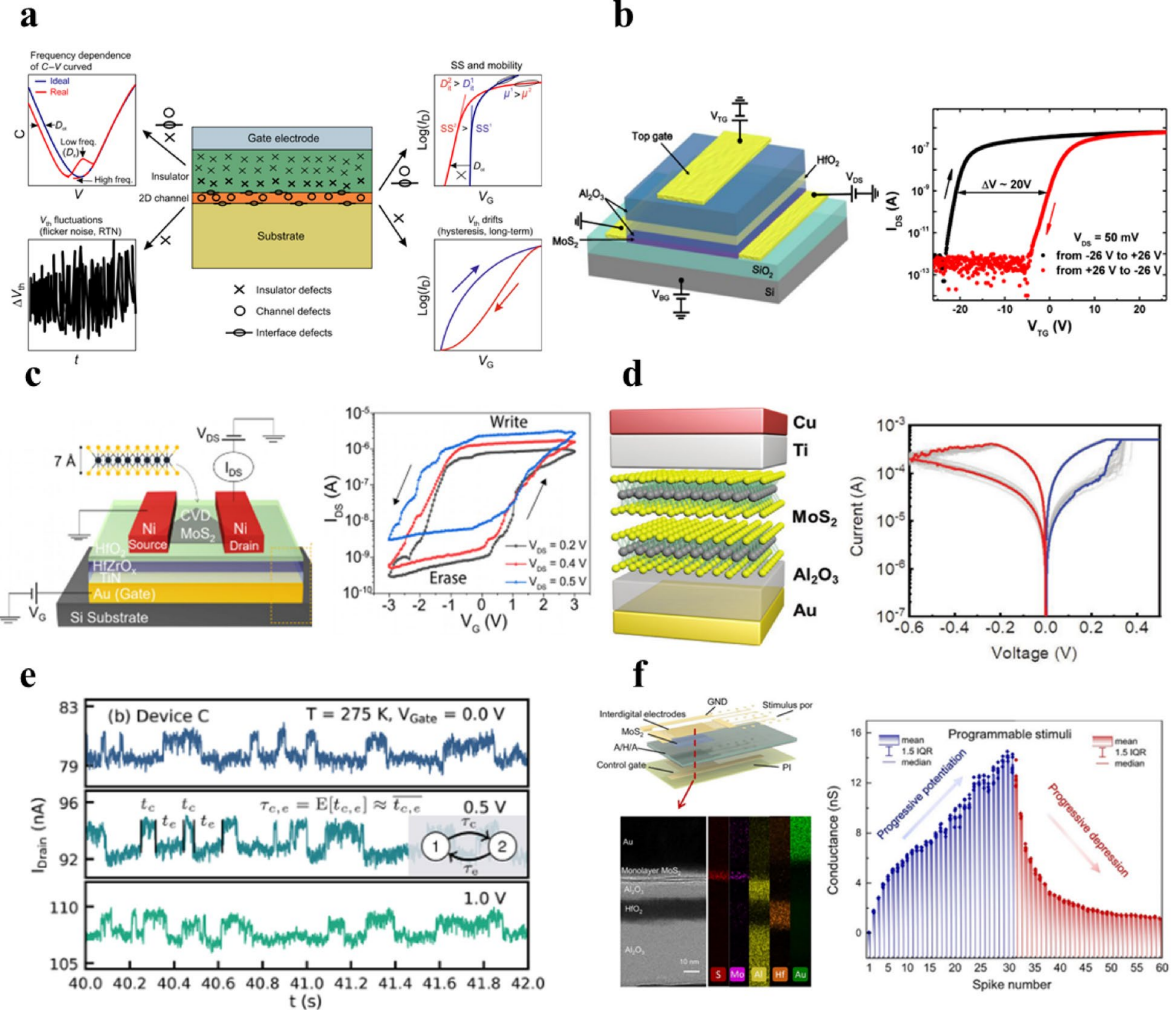
**a** Schematic summary of commonly observed parasitic effects induced by defects in 2D devices [10]. **b** Device structure of tunable charge-trap memory based on few-layer MoS_2_, and a memory window of approximately 20 V from threshold voltage shift is observed during forward and reverse sweeps [72]. **c** Schematic illustration of a single-layer MoS_2_ ferroelectric field-effect transistor incorporating an HZO ferroelectric layer, and its transfer characteristics, demonstrating nonvolatile memory behavior arising from polarization switching [234]. **d** Schematic illustration of an RRAM device structure consisting of a MoS_2_ layer and corresponding I–V characteristics over repeated DC sweeps. The hysteresis in the I–V response implies the formation of a resistive memory window [235]. **e** Random telegraph noise induced by interfacial charge trapping, revealing a key noise mechanism affecting RF transistor performance [177]. **f** Device structure of neuromorphic stimulator based on monolayer MoS_2_ and synaptic potentiation and depression characteristics, emulating long-term potentiation (LTP) and long-term depression (LTD) observed in biological synapses [239]

In logic and low-power transistors, the gate dielectric is typically designed to minimize unwanted components rather than to introduce new functionality. Strong gate coupling and low defect density are required to ensure reliable switching, tight threshold voltage control, and low standby power [42]. From this perspective, charge trapping, hysteresis, and leakage currents are generally viewed as parasitic effects that must be suppressed (Fig. 16a) [10]. Gate stacks optimized for logic operation therefore emphasize interface cleanliness and long-term stability, often at the expense of dielectric scaling.

Memory devices follow a very different design logic. In charge-trap memories, dielectric layers are intentionally used as active storage media, with localized trap states serving as the basis for nonvolatile operation (Fig. 16b) [72]. Ferroelectric field-effect transistors exploit polarization switching and intrinsic hysteresis in ferroelectric dielectrics to encode information in voltage states (Fig. 16c) [234]. Similarly, RRAM relies on defect generation and conductive path formation within dielectric layers to achieve resistive switching (Fig. 16d) [235]. In these architectures, dielectric properties that are problematic for logic devices become central design parameters, and device optimization focuses on controlling defect dynamics rather than eliminating them.

The influence of gate dielectrics is also evident in RF, analog, and optoelectronic devices based on 2D materials, although the trade-offs are often less binary. Dielectric-induced scattering and capacitance affect high-frequency performance in RF transistors, while interfacial charge dynamics can strongly influence gain and noise characteristics (Fig. 16e) [6, 177]. In optoelectronic devices such as photodetectors and sensors, dielectric traps may lead to photogating or persistent photoconductivity. These effects can limit speed and temporal stability, but they are frequently exploited to enhance sensitivity or responsivity, particularly in low-light or sensing applications [236, 237].

Neuromorphic devices represent perhaps the clearest example of how dielectric non-idealities can be reinterpreted as functionality. Gradual charge trapping, hysteresis, and time-dependent conductance changes in dielectric/2D stacks naturally give rise to history-dependent responses that resemble synaptic behavior (Fig. 16f) [238, 239]. Rather than suppressing these effects, neuromorphic architectures often seek to stabilize and tune them, using dielectric dynamics to implement analog weight updates and plasticity [240, 241].

Taken together, these examples underscore that there is no single, universally appropriate dielectric stack for 2D electronics. Instead, dielectric and gate-stack design must align with application-specific priorities, balancing the need for stability and reproducibility with opportunities to harness dielectric-induced effects for memory, sensing, or neuromorphic functionality.

## Challenges and future directions

Despite substantial progress in gate-dielectric integration for 2D electronics, several fundamental challenges remain unresolved and must be addressed before these technologies can meet the requirements of practical manufacturing. This section identifies the key limitations that span the topics discussed in this review and outlines directions that may guide future research.

### Insufficient understanding of ALD nucleation on pristine 2D surfaces

Although surface pretreatments, buffer layers, and process modifications have significantly improved dielectric nucleation on 2D materials, to date, none of these strategies has enabled truly defect-free, conformal growth on pristine dangling-bond-free surfaces without trade-offs [23, 53, 65]. Plasma and UV–O_3_ treatments risk channel oxidation and structural damage, while buffer layers introduce additional series capacitance and can themselves be sources of interfacial traps [99, 111]. The nucleation kinetics on different TMD materials remain incompletely characterized, and the dependence of initial growth modes on channel stoichiometry, defect density, and grain structure is not yet predictive [66, 129]. Developing a unified nucleation strategy universally applicable across materials without compromising intrinsic channel properties remains an open and critical problem.

### Fundamental trade-off between EOT scaling, leakage, and reliability

Aggressive EOT reduction to the sub-nanometer regime inevitably increases direct tunneling, defect-assisted leakage, and dielectric breakdown susceptibility [16, 228, 229]. While some previous research has demonstrated EOT values below 1 nm, these results are frequently reported for individual devices or small arrays, and it remains unclear whether such aggressively scaled stacks can simultaneously satisfy leakage, reliability, and variability targets at wafer scale [19, 99, 102]. The absence of a systematic, cross-study comparison of leakage–EOT–reliability trade-offs across different dielectric materials and integration methods makes it difficult to identify which combinations are genuinely viable for technology nodes beyond 3 nm [40, 230–232]. Nevertheless, establishing fully standardized measurement conditions for EOT, leakage current density, TDDB lifetime, and device-to-device threshold-voltage variation would significantly accelerate progress.

### Immature alternative 2D dielectrics and limited scalability of hBN

As discussed in 2.2, the low dielectric constant of hBN fundamentally limits its use as a standalone gate dielectric in aggressively scaled devices [14, 15]. While emerging high-κ 2D dielectrics such as Bi_2_SeO_5_ and Bi_2_SiO_5_ show promising laboratory-scale results [187, 201, 202], their large-area synthesis, thickness control, and long-term reliability under electrical stress have not been demonstrated at the level required for manufacturing. Similarly, native oxide approaches based on the controlled oxidation of layered semiconductors are compelling in principle [184, 190], but questions remain regarding the reproducibility of the oxidation process, the uniformity of the resulting oxide over wafer-scale areas, and the stability of these oxides under prolonged device operation. Until these materials mature, the field remains heavily reliant on amorphous high-κ oxides whose interface quality with 2D channels is inherently limited [10, 44].

### Wafer-scale uniformity, variability, and manufacturing compatibility

Most of the dielectric integration strategies reviewed here have been demonstrated on exfoliated flakes or small-area CVD films [5, 18]. Translating these results to 300 mm wafer-scale processing introduces additional challenges related to thickness uniformity, grain-boundary effects in polycrystalline TMD films, contamination control, and process-to-process reproducibility. The vdW transfer approach offers a compelling route to decouple dielectric quality from surface reactivity [19, 148, 149], but current implementations rely on sacrificial layers and manual or semi-automated transfer processes whose throughput and yield remain far below CMOS manufacturing standards. Furthermore, the thermal budget constraints imposed by BEOL integration [8, 149] limit the range of post-deposition annealing strategies available to heal dielectric defects [155, 161], creating an additional gap between laboratory-optimized device performance and manufacturable process windows.

### Insufficient understanding of dielectric–channel interactions beyond electrostatics

While the electrostatic effects of gate dielectrics on 2D channels are increasingly well characterized, important secondary effects remain poorly understood. Remote phonon scattering from polar dielectrics [48, 183, 191], dielectric-induced self-heating, strain transfer between oxide and channel [177, 180, 182], and the interplay between charge trapping kinetics and environmental exposure all influence [17, 233] device performance in ways that are difficult to decouple experimentally. Predictive models that capture these coupled effects, particularly under realistic operating conditions with simultaneous electrical, thermal, and environmental stress, are largely absent. Multiscale simulation frameworks that bridge atomistic interface chemistry with device-level transport and reliability would be valuable for guiding dielectric stack optimization.

### Gap between single-device demonstrations and circuit-level validation

The vast majority of dielectric integration studies report performance metrics from individual transistors or small device arrays [5, 7]. However, the viability of a gate-stack technology is ultimately determined by its statistical performance across thousands to millions of devices, where rare outliers in leakage, breakdown, or threshold voltage dominate yield and circuit reliability. Circuit-level demonstrations that incorporate realistic interconnects, thermal management, and multi-device variability analysis remain scarce for 2D electronics [18, 230]. Bridging this gap will require not only improved dielectric processes but also co-design of gate stacks with contacts, interconnects, and encapsulation layers in integrated process flows [2, 6, 8].

## Conclusion

Gate-dielectric integration is emerging as both a primary bottleneck and a key enabler for scalable 2D electronics. Although atomically thin channels offer excellent electrostatics, process-compatible devices ultimately require gate stacks that achieve aggressively scaled EOT while maintaining low leakage, low interfacial trap densities, and sufficient breakdown margins on chemically inert, dangling-bond-free surfaces. The studies reviewed here indicate that interface control is the primary design consideration. Reliable ALD on 2D materials depends on understanding and engineering nucleation, using surface pretreatments and functionalization, and introducing seed or buffer layers that promote conformal growth without introducing excessive damage or trap states. Layered dielectrics, such as hBN, can deliver superior interface quality, but their benefits must be weighed against scaling limits and system-level constraints.

Dielectric selection and processing conditions are closely tied to device stability. Leakage transport, defect generation, hysteresis, threshold-voltage drift, device-to-device variability, and long-term reliability metrics such as TDDB and bias-temperature instability all reflect how defects are created, redistributed, and activated at the dielectric-2D interface. Achieving array-level uniformity targets, therefore, requires process flows and stack designs that are robust against statistical outliers, rather than relying solely on average performance.

Future progress is likely to come from integration-driven approaches that reduce process-induced damage while preserving dielectric quality, including vdW dry integration and dielectric transfer strategies compatible with wafer-scale fabrication. Standardized metrology and reliability protocols tailored to 2D interfaces, combined with co-optimization of dielectric stacks, contacts, and thermal management, will be essential for translating lab-scale demonstrations into scalable platforms. With these developments, gate dielectrics can transition from a bottleneck to a practical pathway toward mixed-dimensional and 3D-stacked architectures that address logic, memory, and emerging functional systems.

## Data Availability

No new data were generated in this study. All relevant information has been obtained from published sources, which are cited in the manuscript.
